# Humoral response induced after intranasal vaccination with heat inactivated *Acinetobacter baumannii* protects immunodeficient mice against hypervirulent LAC-4 strain

**DOI:** 10.3389/fimmu.2025.1641997

**Published:** 2025-09-22

**Authors:** Thomas Rouma, Emeline Barbieux, Lorenzo Bossi, Katy Poncin, Sébastien Denanglaire, Yohannes Tafesse, David Vermijlen, Sandrine Delbauve, Véronique Flamand, Juliette Van Buylaere, Charles Van der Henst, Xavier De Bolle, Fabienne Andris, Eric Muraille

**Affiliations:** ^1^ Unité de Recherche en Biologie des Microorganismes (URBM) - Laboratoire d’Immunologie et de Microbiologie, NAmur Research Institute for LIfe Sciences (NARILIS), University of Namur, Namur, Belgium; ^2^ Laboratoire de Parasitologie, Université Libre de Bruxelles (ULB), Gosselies, Belgium; ^3^ ULB Center for Research in Immunology (U-CRI), Gosselies, Belgium; ^4^ ImmunXperts SA, a Q² Solutions Company, Gosselies, Belgium; ^5^ Immunobiology Laboratory, ULB, Gosselies, Belgium; ^6^ Institute for Medical Immunology, ULB, Gosselies, Belgium; ^7^ Department of Pharmacotherapy and Pharmaceutics, ULB, Gosselies, Belgium; ^8^ Microbial Resistance and Drug Discovery, VIB-VUB Center for Structural Biology, VIB, Flanders Institute for Biotechnology, Brussels, Belgium; ^9^ Structural Biology Brussels, Vrije Universiteit Brussel (VUB), Brussels, Belgium

**Keywords:** *Acinetobacter baumannii*, LAC-4 strain, pulmonary infection, whole body inactivated vaccine, immunodeficient mice, peripheral blood mononuclear cells of patients

## Abstract

**Background:**

*Acinetobacter baumannii* is an opportunistic bacterium that causes serious nosocomial infections, including pneumonia and bacteremia, especially in immunocompromised individuals.

**Methods:**

Here, we first evaluated the protective efficacy of a vaccination protocol with the heat-killed (HK) *A. baumannii* strain LAC-4 in various models of immunodeficient C57BL/6 mice challenged with pulmonary infection by LAC-4. We then examined the ability of HK LAC-4 to stimulate peripheral blood mononuclear cells (PBMCs) from healthy donors *in vitro*.

**Results:**

We observed that mice deficient in Th1 (IL-12p35^-/-^, IFN-γ^-/-^), Th17 (IL-17RA^-/-^) and T cells (δTCR^-/-^, TAP1^-/-^, CD3^-/-^) display higher susceptibility to LAC-4 infection, but that our protocol improves their resistance. In contrast, vaccinated B cell-deficient (MuMT^-/-^) mice appear unable to control the infection, demonstrating that humoral immunity is essential to vaccine protection. Vaccination of wild-type mice with an HK Δ*itrA* strain deficient for capsule production failed to induce protection, showing that protective antibodies are mainly directed against the capsule. Our vaccination protocol also confers increased protection in wild-type mice vaccinated and then treated with cyclophosphamide; an immunosuppressive drug described to strongly increase the susceptibility of mice to *A. baumannii* infection. Finally, we demonstrate that HK LAC-4 can induce the activation of human monocyte-derived dendritic cells and T lymphocytes from PBMCs of healthy donors, suggesting that it may activate the human adaptive immune system and induce a protective memory response against *A. baumannii*.

**Conclusions:**

Overall, our results demonstrate that administration of HK bacteria can induce protective immunity against *A. baumannii* in both immuno-competent and immuno-compromised mice and that these HK bacteria can activate the human adaptive immune system.

## 1 Introduction

*Acinetobacter baumannii* is an aerobic Gram-negative coccobacillus and an opportunistic nosocomial pathogen (1, 2). It is responsible for a wide range of human infections, including ventilator-associated pneumonia, bloodstream infections, wound and burn infections, urinary tract infections, and meningitis. Prolonged hospital stays—especially in intensive care units (ICU)—mechanical ventilation, indwelling foreign devices, extended exposure to antimicrobial treatment and immunodepression have been linked to multidrug-resistant *A. baumannii* infections (3). A relatively recent development is the association of *A. baumannii* with infections following injuries sustained in conflict zones, such as those in Iraq and Afghanistan (4, 5). Some evidence suggests that the use of morphine — commonly administered after battlefield or crush injuries — may enhance *A. baumannii* infections, possibly due to its immunosuppressive effects (6).

The genome of *A. baumannii* presents great diversity, with 2,119 genes forming the core genome and > 16,000 the pan genome (7), and high plasticity due to a very high rate of horizontal gene transfer (8). As a consequence, *A. baumannii* has an extraordinary ability to rapidly acquire resistance to antibiotics (9). Multidrug resistant strains (MDR) of *A. baumannii* have been documented worldwide. Several isolates are resistant to all commercially available antibiotics (7, 10). *A. baumannii* produces a high molecular weight capsular polysaccharide (11) which surrounds the outer membrane and makes it highly resistant to desiccation, disinfectants (12, 13) and reactive oxygen species (14). Significant variation in capsular polysaccharide structures between *A. baumannii* isolates has been identified (11), with several hundred distinct capsule types, incorporating a vast variety of sugars (15). *A. baumannii* is also able to form biofilms on biotic and abiotic surfaces, which further increases its survival on medical equipment as well as its resistance to detergents (9).

These characteristics make *A. baumannii* a pathogen particularly well suited to the hospital environment and a danger to patients hospitalized for long periods. Recently, the incidence of MDR *A. baumannii* co-infection has increased in intensive care units due to prolonged hospitalization of COVID-19 patients (16, 17). For example, among hospitalized COVID-19 patients in Wuhan, China, nearly half of the individuals who developed secondary bacterial infections died, and *A. baumannii* accounted for 36% of those infections (18). This phenomenon could partly be a consequence of the administration of high doses of dexamethasone, a steroid well known for its immunosuppressive effects, to treat patients with Covid-19 (19).

Consequently, the *World Health Organization* has defined carbapenem-resistant *A. baumannii* as a Priority 1 ‘‘critical’’ organism, and ranked it the number 1 organism recommended for new research and development (20). It has also been placed on the ESKAPE Restricted Pathogen List and ranked as a top priority by the *US Centers for Disease Control and Prevention* (21).

Vaccination is one of the most effective measures for infection control and is likely to be unaffected by the drug resistance of the pathogen. However, the development of a vaccine against *A. baumannii* has been plagued with several specific problems. The great variability of the *A. baumannii* genome (8) and capsule (11, 15) makes the development of a subunit vaccine focusing the adaptive response on a small number of antigenic determinants risky. Moreover, little is known about the protective immune response to *A. baumannii*, which limits our ability to identify immune markers for selecting vaccine candidates based on the responses they elicit. Studies in mouse models show that intranasal inoculation of *A. baumannii* induces high production of pro-inflammatory cytokines and rapid recruitment of neutrophils to the lungs. Several pattern recognition receptors, such as TLR4 (22), TLR9 (23) and NOD2 (24) have been implicated in *A. baumannii* detection and control, suggesting that a rapid response to infection is critical to its control. Complement, macrophages and neutrophils have been demonstrated to play a key role (25, 26). On the other hand, *A. baumannii* possesses several virulence mechanisms that allow it to escape the immune system and resist environmental stress. In particular, its capsule plays an important role in protection against destruction by host cells and escape from the innate immune response (27, 28). In particular, very little is known about the protective adaptive response against *A. baumannii* infection. The role of Th1 and Th17 responses, as well as the importance of the humoral and cellular response, have been the subject of debate (29). Furthermore, in some mouse models of infection, the animal does not develop a protective memory response (30).

The *A. baumannii* strain LAC-4 (31) was clinically isolated from a nosocomial outbreak in Los Angeles County hospitals in 1997 (32). In mouse models, LAC-4 intranasal infections reliably reproduce the most relevant features of human pulmonary *A. baumannii* infection and the corresponding pathology. Within 24 hours post-infection, LAC-4 displays rapid bacterial replication in the lungs, significant extrapulmonary dissemination to the spleen and severe bacteremia (33). Intranasal immunization of wild-type mice with formalin-killed whole LAC-4 bacteria has been shown to induce the development of short-term immunity via training of alveolar macrophages (34) and long-term immunity against intranasal challenge with live LAC-4 (35).

In the present study, we attempted to determine whether a single intranasal vaccination with the heat killed LAC-4 strain protects mice genetically deficient for key elements of the immune response as well as mice treated with cyclophosphamide, an immunosuppressive drug frequently used in clinical settings to treat cancers and autoimmune diseases (36) and described to greatly increase susceptibility to infections by *A. baumannii* in mice (37). We also attempted to determine whether killed LAC-4 can induce activation of human dendritic cells and T lymphocytes.

## 2 Results

### 2.1 Immunization with a killed strain of AB5075 or LAC-4 induces protection against challenge with a live strain of *A. baumannii*

The LAC-4 (33) and AB5075 (38) strains of *A. baumannii* have previously been reported as highly virulent in mouse models and reproducing the main features of the pathology of a human pulmonary infection by *A. baumannii*. Interestingly, these strains are not closely related. They differ substantially in allelic variants of housekeeping genes and do not qualify as single- or double-locus variants of each other under either the Pasteur or Oxford schemes. LAC-4 is classified as Sequence Type (ST)10 and has not been assigned to any Global Clone (31). LAC-4 is sometimes referred to as Clonal Complex (CC)10, whereas AB5075 is classified as ST1 and belongs to Clonal Group I (39).

In our C57BL/6 intranasal infection model, we found that AB5075 exhibits lower virulence than LAC-4. Bacterial loads in the lungs at 24 hours post-infection were higher for LAC-4 (**Supplementary Figure S1A**), and this strain induced significant mortality at a dose of 2×10^7^ CFU, whereas all mice survived the same dose of AB5075 (**Supplementary Figure S2B**). Therefore, we choose to use strain LAC-4 to test the acquisition of protective memory against *A. baumannii* infection.

A vaccine composed of 5x10^5^ or 5x10^7^ CFU of formalin-killed *A. baumannii* LAC-4 administered intranasally has been shown (35) to fully protect wild-type C57BL/6 mice against a challenge with 7x10^7^ CFU of live LAC-4 that killed 80% of control unvaccinated mice. We choose to use a similar experimental model with a vaccine composed of heat-killed (HK) *A. baumannii* and tested whether intranasal or intraperitoneal administration of HK *A. baumannii* LAC-4 or AB5075 strains could induce long-lasting immunity against a semi lethal challenge with the live LAC-4 strain.

**Figure 1:**
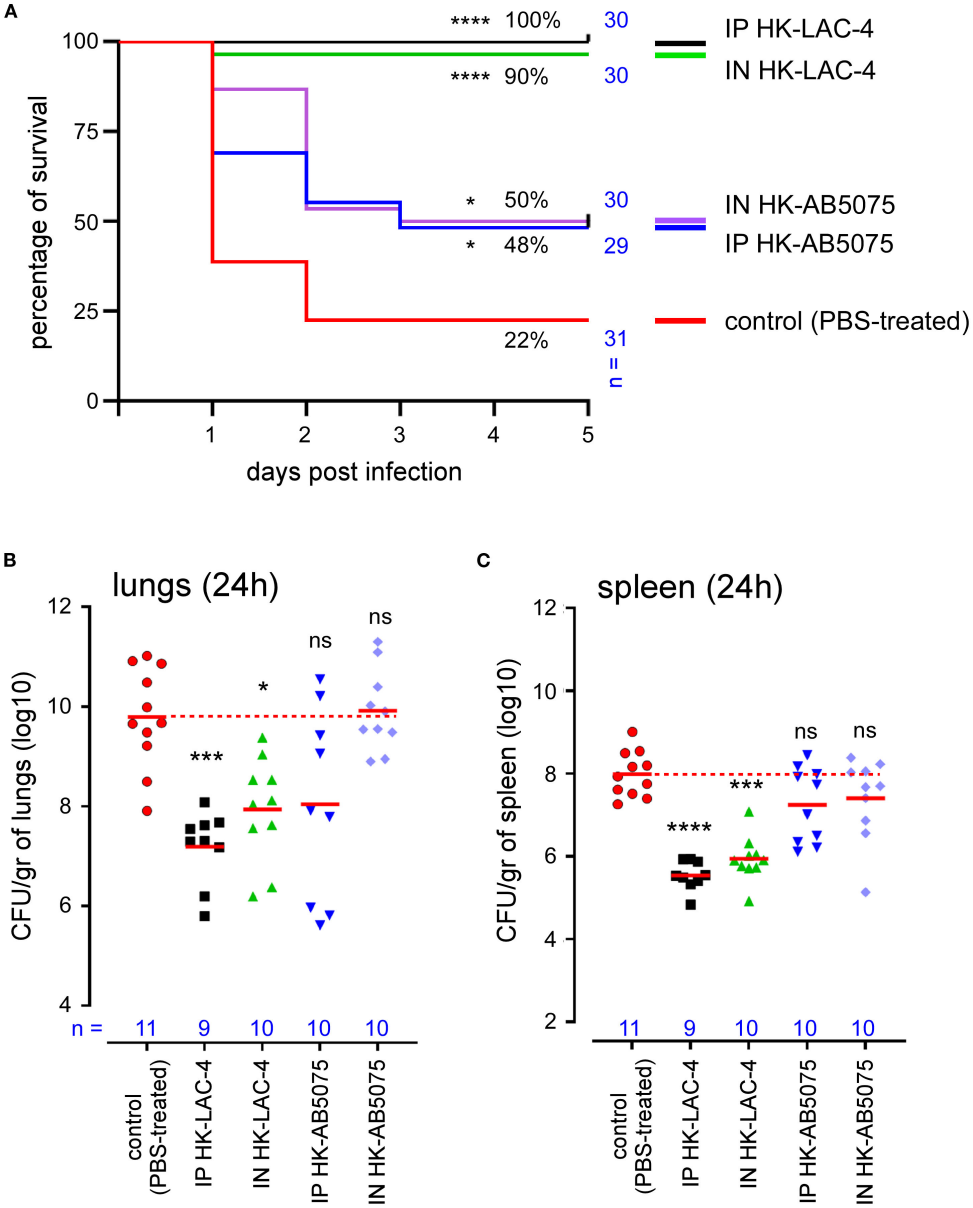
Intranasal immunization with heat-killed LAC-4 or AB5075 *A. baumannii* strains confer significant protection against challenge with live LAC-4 strain. Wild-type C57BL/6 mice were inoculated intranasally (IN) or intraperitoneally (IP) with PBS (control) or 10^8^ CFU of heat-killed (HK) LAC-4 or HK 5075 A*. baumannii* strains in PBS. 6 weeks later, control and vaccinated mice were challenged intranasally with 2x10^7^ CFU of live LAC-4 strain. **(A)** Survival rate. Over the 5 days after challenge, the fitness of infected mice was monitored. When the human endpoint was reached, the mice were euthanized. These data represent a pool of two independent experiments. n = number of mice per group. **(B, C)** CFU counts. 24 hours after challenge, some mice were sacrificed and the number of bacteria per gram in the lungs **(B)** and spleen **(C)** was evaluated by CFU counting. Red lines represent the geometric mean for each group of mice. Dotted red line represents the mean value of the control (unvaccinated) group. Significant differences between control (unvaccinated) and vaccinated groups are marked with asterisks: *p < 0.05, ***p < 0.001, ****p < 0.0001, in a log-rank (Mantel–Cox) test for survival curve and a (Wilcoxon-) Mann-Whitney post-test for CFU count.

Wild-type C57BL/6 mice were inoculated intranasally or intraperitoneally with a solution of PBS (control unvaccinated group) or 10^8^ CFU of HK AB5075 or HK LAC-4 in PBS. Six weeks later, 2x10^7^ CFU of live LAC-4 was administered intranasally to all groups. Survival was evaluated in each group of mice up to 5 days post-challenge (**Figure 1A**). The bacteria count in the lungs (**Figure 1B**) and spleen (**Figure 1C**) of the mice was measured by counting the CFUs at 24 hours post-challenge.

We observed that all groups immunized with HK LAC-4 or HK AB5075 showed significantly improved survival compared to the unvaccinated controls (**Figure 1A**). As expected, immunization with LAC-4 provided stronger protection against LAC-4 infection than AB5075 immunization. Nevertheless, AB5075 immunization still conferred partial but statistically significant protection against LAC-4, indicating that vaccination with one strain of *A. baumannii* can induce cross-protection against genetically distant strains. Survival of HK LAC-4 groups correlated with bacterial control in both the lungs (**Figure 1B**) and spleen (**Figure 1C**). Interestingly, this is not the case with the protection conferred by HK AB5075, suggesting that survival is driven more by the nature of the inflammatory response than by the absolute bacterial burden in the organs.

### 2.2 Intranasal immunization with heat-killed LAC-4 induces cross protective *A. baumannii* antibodies and robust production of proinflammatory cytokines

**Figure 2:**
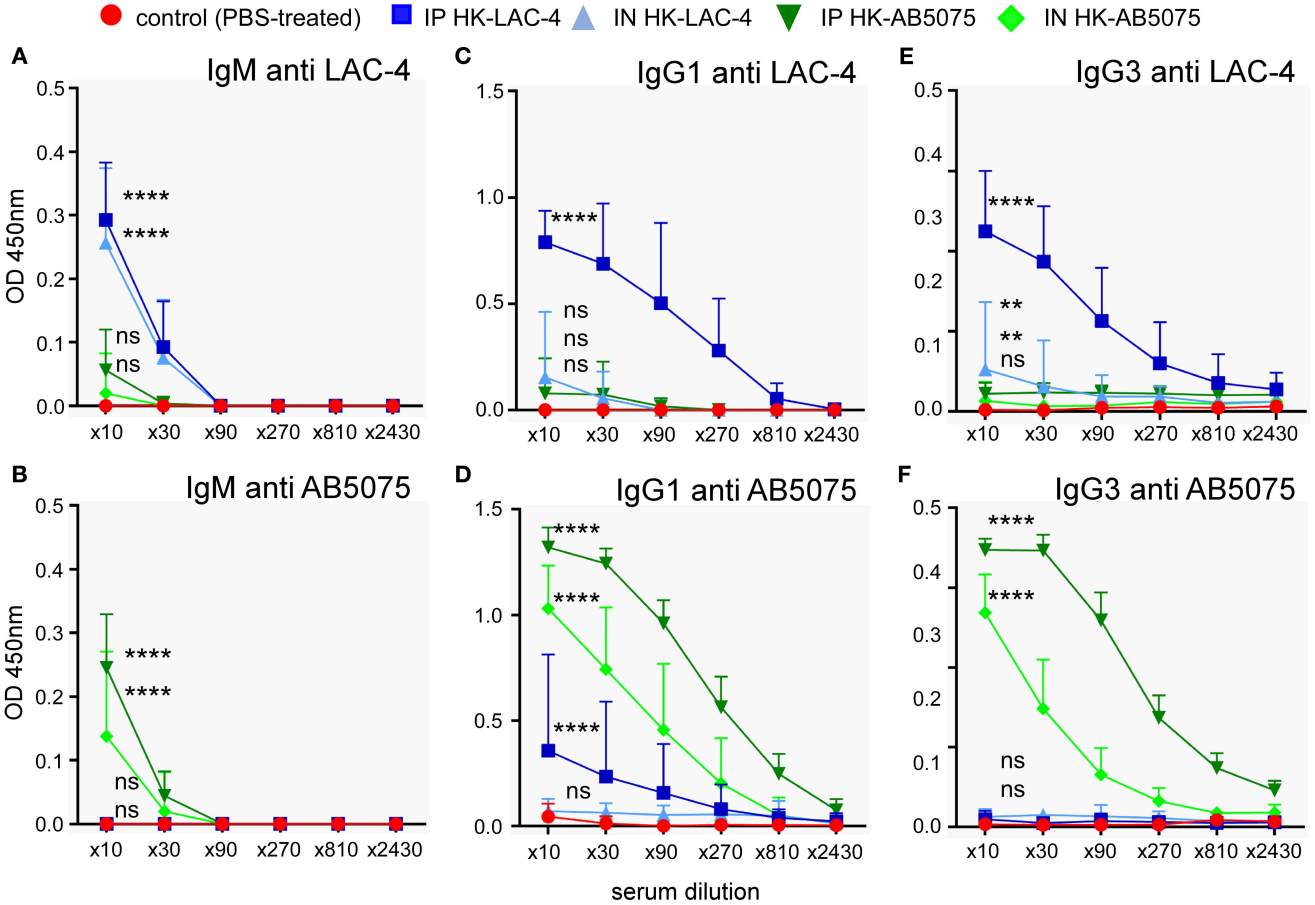
Humoral immune response induced by intranasal immunization with heat-killed *A. baumannii*. Wild-type C57BL/6 mice were inoculated intranasally with 10^8^ CFU of heat-killed (HK) strains of *Acinetobacter baumannii* (LAC-4 in blue or AB5075 in green), as indicated. Serum was collected at 4 weeks post-vaccination, and ELISA was performed to determine the levels of IgM, IgG1 and IgG3 antibodies specific to LAC-4 **(A, C, E)** and AB5075 **(B, D, F)**. The data represent the means ± SD of 8 mice per group. O.D., optical density. Significant differences between control (unvaccinated) and vaccinated groups are marked with asterisks: **p < 0.01, ****p < 0.0001, in a two-way Anova with Dunnett’s multiple comparisons test.

Since protection against *A. baumannii* in mice vaccinated with formalin-killed LAC-4 was attributed to the presence of specific antibodies (35), we measured the presence of antibodies specific for LAC-4 and AB5075 in the serum of mice vaccinated intraperitoneally or intranasally with LAC-4 or AB5075. Our results show that immunization via the intraperitoneal route induces higher levels of specific IgM, IgG1 and IgG3 antibodies than immunization via the intranasal route (**Figure 2**). As expected, immunization induces a higher level of antibodies against the vaccination strain compared to the other strain. Nevertheless, immunization with LAC-4 induces a low but statistically significant level of antibodies able to recognize AB5075 and vice versa, which may explain the moderate cross protection we observed following a challenge with LAC-4.

**Figure 3:**
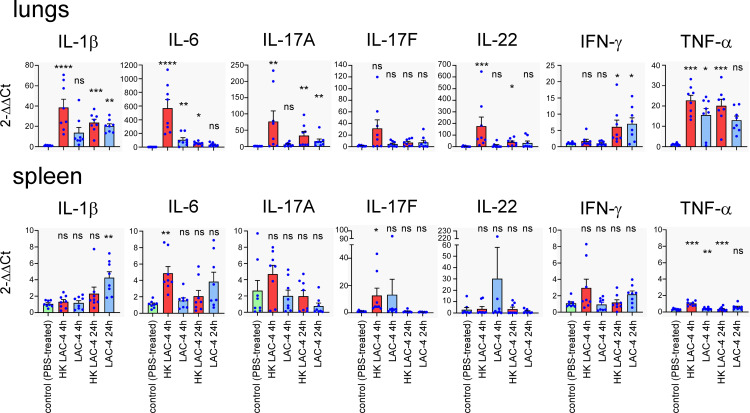
Intranasal administration of heat-killed *A. baumannii* LAC-4 strain induces the production of proinflammatory cytokines in the lungs and spleen. Wild-type C57BL/6 mice were inoculated intranasally with PBS (control), 10^8^ CFU of heat-killed (HK) or 2x10^6^ CFU of live *A. baumannii* LAC-4 strain in PBS, as indicated. At 4 and 24 hours post-infection, mice were sacrificed, lungs and spleen were collected. Expression levels of the indicated genes were assessed by quantitative RT-PCR and expressed as relative expression to RPL32 housekeeping mRNA. The naïve mice (number 1) condition was set to 1. The experiment was performed twice independently with 4 mice per group. The figure represents a pool of data from the 2 experiments, n=8 mice for each condition. Significant differences between control (unvaccinated) and vaccinated groups are marked with asterisks: *p < 0.05, **p < 0.01, **p < 0.001, ***p < 0.001, in a (Wilcoxon-) Mann-Whitney post-test.

To better understand the nature of the protection conferred by the HK LAC-4 vaccine, we compared the production of proinflammatory cytokines induced by an intranasal infection with 2x10^6^ CFU of live LAC-4 and the intranasal administration of 10^8^ CFU of HK LAC-4. We administered a lower dose of live LAC-4 than dead LAC-4 because mice could not survive 24 hours after injection of 10^8^ CFU of live LAC-4. The levels of various cytokines were measured at 4 and 24 hours post-intranasal inoculation in the lungs and the spleen by q-PCR reaction (**Figure 3**). We observed that HK LAC-4 induces innate proinflammatory cytokines e.g. IL-1, IL-6, TNF-α, as well as cytokines associated with both Th1 (IFN-γ) and Th17 (IL-17A, IL-17F and IL-22) responses. However, compared with live LAC-4, HK LAC-4 induces less IFN-γ but more IL-17F and IL-22, suggesting that HK LAC-4 would be particularly effective in inducing Th17 immunity.

### 2.3 Mice rendered genetically deficient in IL-17RA dependent response or γδT cells are highly susceptible to pulmonary *A. baumannii* infection

To determine which elements of the adaptive immune response are essential for control of an intranasal infection, we studied the survival of different strains of mice deficient for key elements of the immune system following infection with LAC-4.

**Figure 4:**
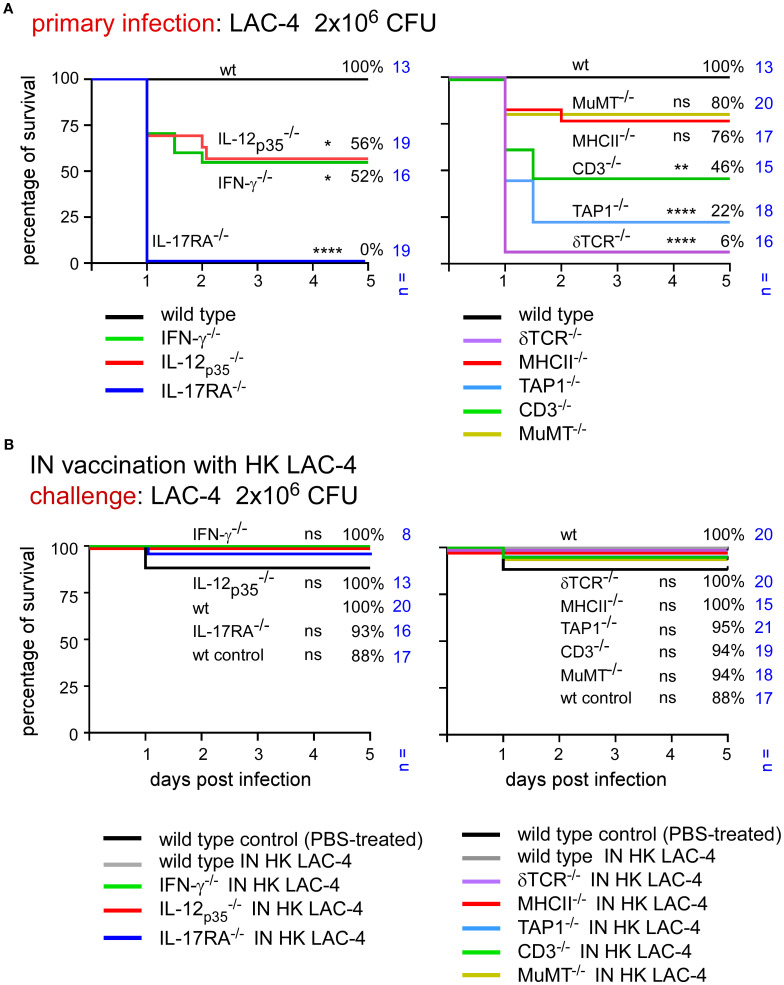
Immunization with HK LAC-4 protects wild-type mice and mice deficient for key elements of adaptive immune response against LAC-4 challenge. The figures present survival rates of wild-type and genetically deficient C57BL/6 mice measured over a period of 5 days after intranasal infection with *A*. *baumannii* LAC-4. When the human endpoint was reached, the mice were euthanized. **(A)** Mice were infected with 2x10^6^ CFU of LAC-4. **(B)** Mice were treated intranasally with PBS (control) or 10^8^ CFU of heat-killed (HK) LAC-4 in PBS. 6 weeks later, control and vaccinated mice were challenged intranasally with 2x10^6^ CFU of live LAC-4. The data represent the pool of a minimum of two distinct experiments. n indicates the number of mice per group. Significant differences between wild-type mice **(A)** or vaccinated wild-type **(B)** and other groups are marked with asterisks: *p < 0.05, **p < 0.01, ****p < 0.0001, in a log-rank (Mantel–Cox) test.

Wild-type mice as well as mice rendered genetically deficient for the Th1 response (IL-12p35^-/-^, IFN-γ-/-), for the Th17 signaling (IL-17RA^-/-^) and for selected lymphocyte populations (δTCR^-/-^, TAP-1^-/-^, MHCII^-/-^, CD3^-/-^, MuMT^-/-^) were infected intranasally with 2x10^6^ CFU of live LAC-4. Survival was measured in each group for 120 hours post-infection (**Figure 4A**). We observed that the absence of IL-17RA and δTCR has dramatic consequences. Almost all mice died within 24 hours in these groups (0 and 6% survival in the 17RA- and δTCR-KO groups, respectively). The levels of IL-17A, IL-17F, IL-22 and TNF-α were measured at 4 and 24 hours post-intranasal inoculation in the lungs of wild-type and δTCR^-/-^ mice by q-PCR reaction (**Supplementary Figure S2**). We observed that mice deficient for δTCR do not produce fewer mRNA for these cytokines than wild-type mice, which suggests that the absence of γδ+T cells does not impair the production of these cytokines. A deficiency in TAP1 also induces significant early mortality with only 22% survival. CD3 deficiency (which affects all T cells) is associated with 46% survival of mice, while MHCII deficiency [which affects CD4^+^ T cells (40)] and MuMT [which affects B cells (41)] allows 76% and 80% survival, respectively. A statistical analysis of these results shows that the absence of CD3 significantly reduces the survival of the animals, whereas the deficiency of MHC-II or MuMT has no significant effects on it.

Surprisingly, the CFU counts observed in the lungs and spleen of infected mice weakly correlated with survival of the mouse (**Supplementary Figure S3**). All groups of deficient mice had a higher level of bacteria in the lungs and spleen than wild-type mice and the differences were quite small, particularly in spleen, between the deficient mice. These results suggest that it is the nature of the inflammatory immune response rather than the number of bacteria in organs that determines the animal’s survival.

Taken together, these results suggest that γδ T cells, TAP-1 dependent lymphocyte populations and IL-17RA-dependent signaling play a very important role in the control of primary intranasal LAC-4 infection while the role of IL-12 and the IFN-γ dependent Th1 response as well as CD4^+^ T cells and B cells is more moderate.

### 2.4 Intranasal immunization with heat-killed LAC-4 induces long-term immune protection in a large panel of mice genetically deficient for key elements of the adaptive immune response

After identifying the genetic deficiencies leading to increased susceptibility to primary infection, we tested the capacity of intranasal immunization with HK LAC-4 to increase the survival of genetically immunodeficient mice against pulmonary LAC-4 infection.

Wild-type mice were inoculated intranasally with PBS (control, unvaccinated group) or 10^8^ CFU of HK LAC-4 in PBS. The survival of these mice was compared with that of various genetically immunodeficient mice vaccinated with HK LAC-4 and subjected to an intranasal challenge with a dose of 2x10^6^ CFU of live LAC-4 (**Figure 4B**). We observed that the survival rates in all groups of vaccinated immunodeficient mice were high (88-100%). This suggests that vaccination compensates for the absence of the Th1 or Th17 response as well as the TAP-1 and CD3 deficiencies.

**Figure 5:**
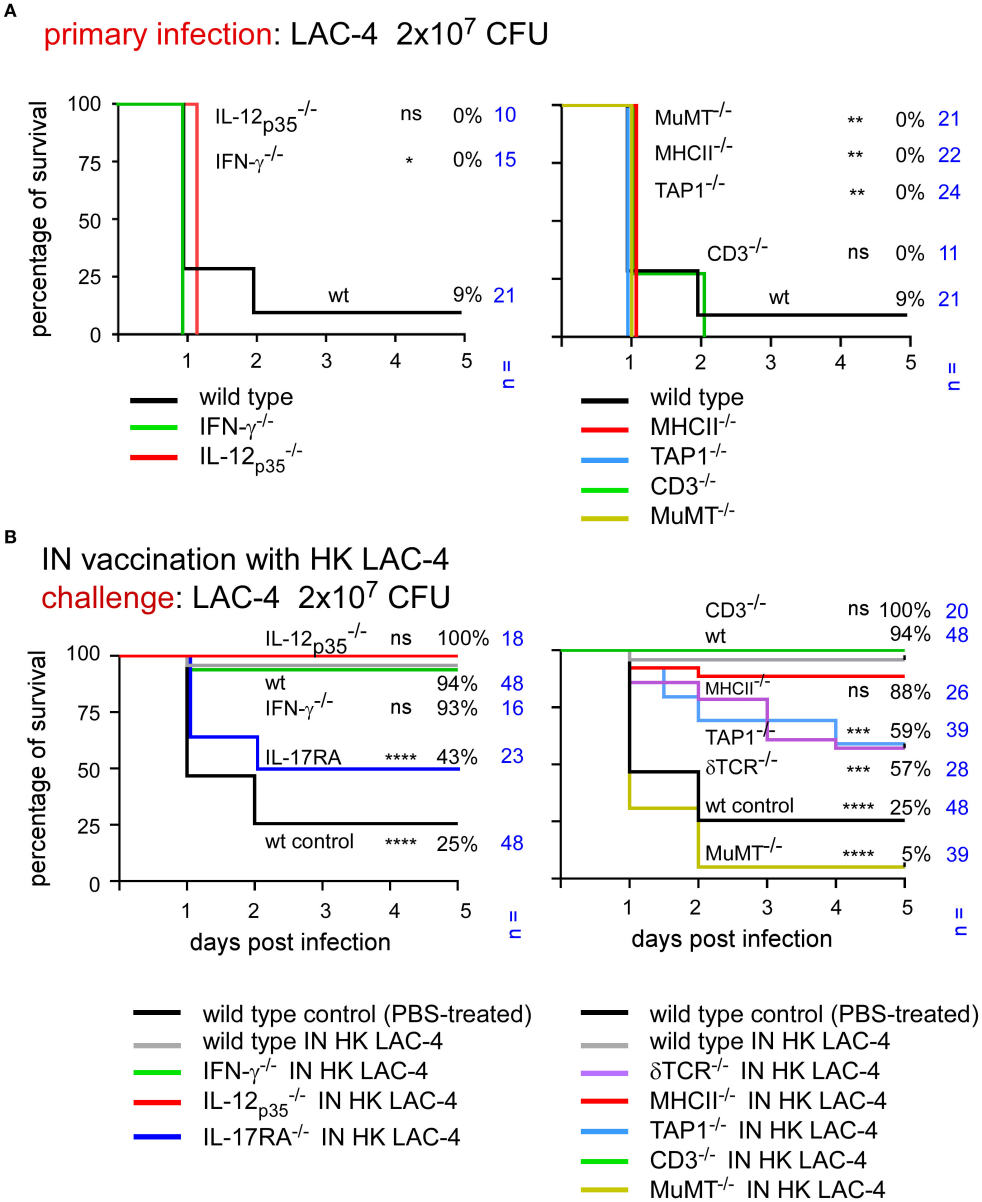
Immunization with HK LAC-4 fails to protect some deficient mice against high-dose LAC-4 challenge. The figures present the survival rates of wild-type and genetically deficient C57BL/6 mice measured over a period of 5 days after intranasal infection with *A*. *baumannii* LAC-4. When the human endpoint was reached, the mice were euthanized. **(A)** Mice were infected with 2x10^7^ CFU of LAC-4. **(B)** Mice were treated intranasally with PBS (control) or 10^8^ CFU of heat-killed (HK) LAC-4 in PBS. 6 weeks later, control and vaccinated mice were challenged intranasally with 2x10^7^ CFU of live LAC-4. The data represent the pool of a minimum of two distinct experiments. n indicates the number of mice per group. Significant differences between wild-type mice **(A)** or vaccinated wild-type **(B)** and other groups are marked with asterisks: *p < 0.05, **p < 0.01, ***p < 0.001, ****p < 0.0001, in a log-rank (Mantel–Cox) test.

To identify the key elements of protective memory immunity induced by HK LAC-4, we challenged vaccinated mice with a higher dose of live LAC-4, 2x10^7^ CFU. At this dose, most unvaccinated mice, whether wild-type or genetically immunodeficient, succumb to infection within 48 hours (**Figure 5A**). Interestingly, the vaccine does not provide equivalent protection against a challenge with 2x10^7^ CFU of live LAC-4 in all groups of immunodeficient mice (**Figure 5B**). MuMT^-/-^ mice showed the lowest survival rate, at 5%, thus confirming the key role of antibodies in protective memory against *A. baumannii*. Vaccinated TAP1^-/-^, δTCR^-/-^ and IL-17A^-/-^mice also show statistically significant reduction of survival, at 59%, 57% and 43% respectively, compared to vaccinated wild-type mice (94%). These findings demonstrate that vaccination cannot completely compensate for the absence of cell populations that depend on TAP1 and δTCR or of the IL-17RA-dependent Th17 response. In contrast, vaccine protection can be induced in the absence of CD3 or MHCII-dependent T cells as well as the IL-12_p35_ or IFN-γ dependent Th1 response.

### 2.5 The bacterial capsule is essential for the induction of protective memory

Since the humoral response is a key element of protection against *A. baumannii* infection induced by HK LAC-4 in our experimental model, we attempted to identify the main antigenic targets of the protective antibodies. To this end, we produced a LAC-4 strain deficient for the *itrA* gene (as described in the Materials and Methods section), which is essential for bacterial capsule synthesis (42), and compared the vaccine capacity of this ΔitrA strain to that of the wild-type strain.

**Figure 6:**
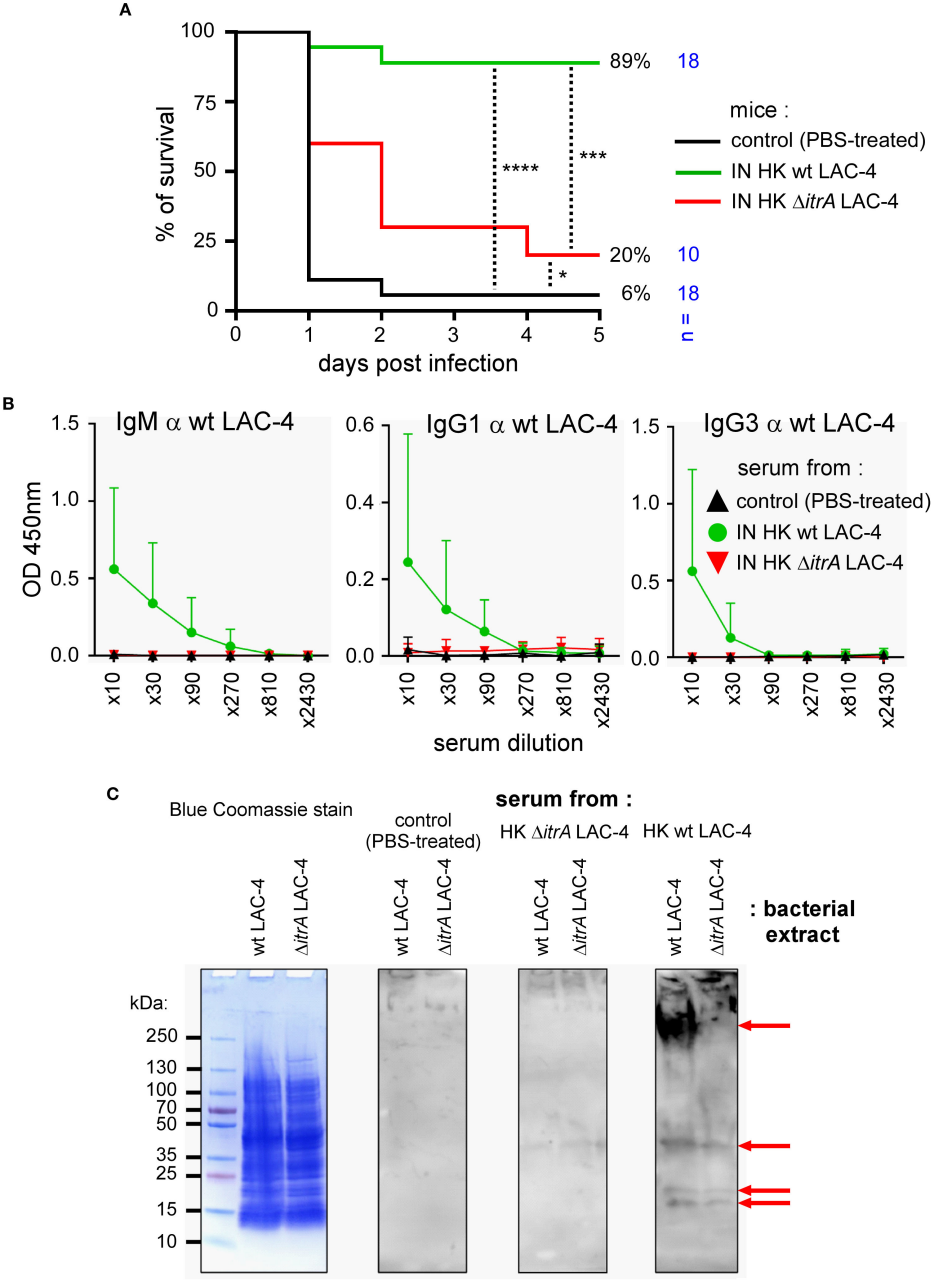
Vaccine produced with capsule-deficient bacteria fails to induce protection in mice. C57BL/6 mice were treated intranasally (IN) with PBS (control) or 10^8^ CFU of heat-killed (HK) of wild-type LAC-4 or ΔitrA LAC-4 in PBS. **(A)** At 6 weeks post-vaccination, control and HK vaccinated mice were challenged intranasally with 2x10^7^ CFU of live wild-type LAC-4 strain. The figure presents the survival rates of each group of mice measured over a period of 5 days after intranasal infection with *A*. *baumannii* LAC-4. When the human endpoint was reached, the mice were euthanized. The data represent the pool of two distinct experiments. n indicates the number of mice per group. **(B)** At 4 weeks post-vaccination, serum was collected, and ELISA was performed to determine the levels of IgM, IgG1 and IgG3 antibodies specific to LAC-4, as indicated. The data represent the means ± SD of 8 mice per group. O.D., optical density. **(C)** Antibodies produced by vaccinated mice were tested against bacterial samples of capsulated wild-type and non-capsulated Δ*itrA* LAC-4 strains. Pool of serums from unvaccinated mice was used for background control, while Coomassie Blue stain was used as loading control and to visualize proteinaceous content. Pool of serums from wild-type LAC-4 vaccinated mice targeted high molecular weight antigens in the capsulated strains only, as well as conserved lower molecular weight proteins in both strains. These results are representative of 3 independent experiments. Significant differences between groups **(A)** are marked with asterisks: *p < 0.05, ***p < 0.001, ****p < 0.0001, in a log-rank (Mantel–Cox) test.

Wild-type C57BL/6 mice were vaccinated intranasally with 10^8^ CFU of HK wild-type or ΔitrA LAC-4 strains. At 6 weeks post-vaccination, mice were infected with 2x10^7^ CFU of live wild-type LAC-4 strain. As expected, most mice vaccinated with wild-type HK LAC-4 survived (89%) (**Figure 6A**). In striking contrast, mice vaccinated with HK ΔitrA LAC-4 had a very low survival rate (20%), close to that of unvaccinated mice (6%). Measurement by ELISA of the levels of antibodies specific to wild-type LAC-4 in the blood of vaccinated animals showed that mice vaccinated with HK ΔitrA did not have IgM, IgG1 and IgG3 antibodies recognizing wild-type LAC-4 (**Figure 6B**).

To better identify the targets of protective antibodies against LAC-4 infection, we conducted Western blot analyses (**Figure 6C**) comparing antigens recognized by sera from mice vaccinated with PBS (control), wild-type, or capsule-deficient ΔitrA LAC-4 strains. Extracts from wild-type LAC-4 and Δ*itrA* LAC-4 were tested. Sera from mice immunized with wild-type LAC-4 recognized a large count of high–molecular-weight antigens in extract from wild-type LAC-4, whereas no corresponding signal was detected in capsule-deficient Δ*itrA* LAC-4 extract. This intense signal is not revealed by Coomassie blue staining. We also detected with serum from mice vaccinated with wild-type LAC-4 strains the presence of low molecular weight antigens in both extracts that are visible in Coomassie blue. Overall, these results demonstrate that protective antibodies induced by vaccination with HK wild-type LAC-4 are directed against non-proteinaceous capsule antigens and against some low molecular weight protein antigens.

### 2.6 Intranasal immunization with heat-killed LAC-4 partially protects cyclophosphamide-treated mice against challenge with *A. baumannii*

Since hospitalized patients at risk for *A. baumannii* infection are often immunocompromised (9), we tested the ability of intranasal vaccination with HK LAC-4 to protect cyclophosphamide-treated mice against intranasal infection with LAC-4. Wild-type C57BL/6 mice were inoculated intranasally with PBS (control) or 10^8^ CFU of HK LAC-4. Five weeks later, half of the mice in each group were inoculated intraperitoneally with PBS or underwent cyclophosphamide treatment, as described in the Materials and Methods. One week post- cyclophosphamide treatment (6 weeks post-vaccination), all groups of mice received 2×10^6^ CFU of live LAC-4 intranasally. Survival of the mice was measured up to 120 hours post-challenge.

**Figure 7:**
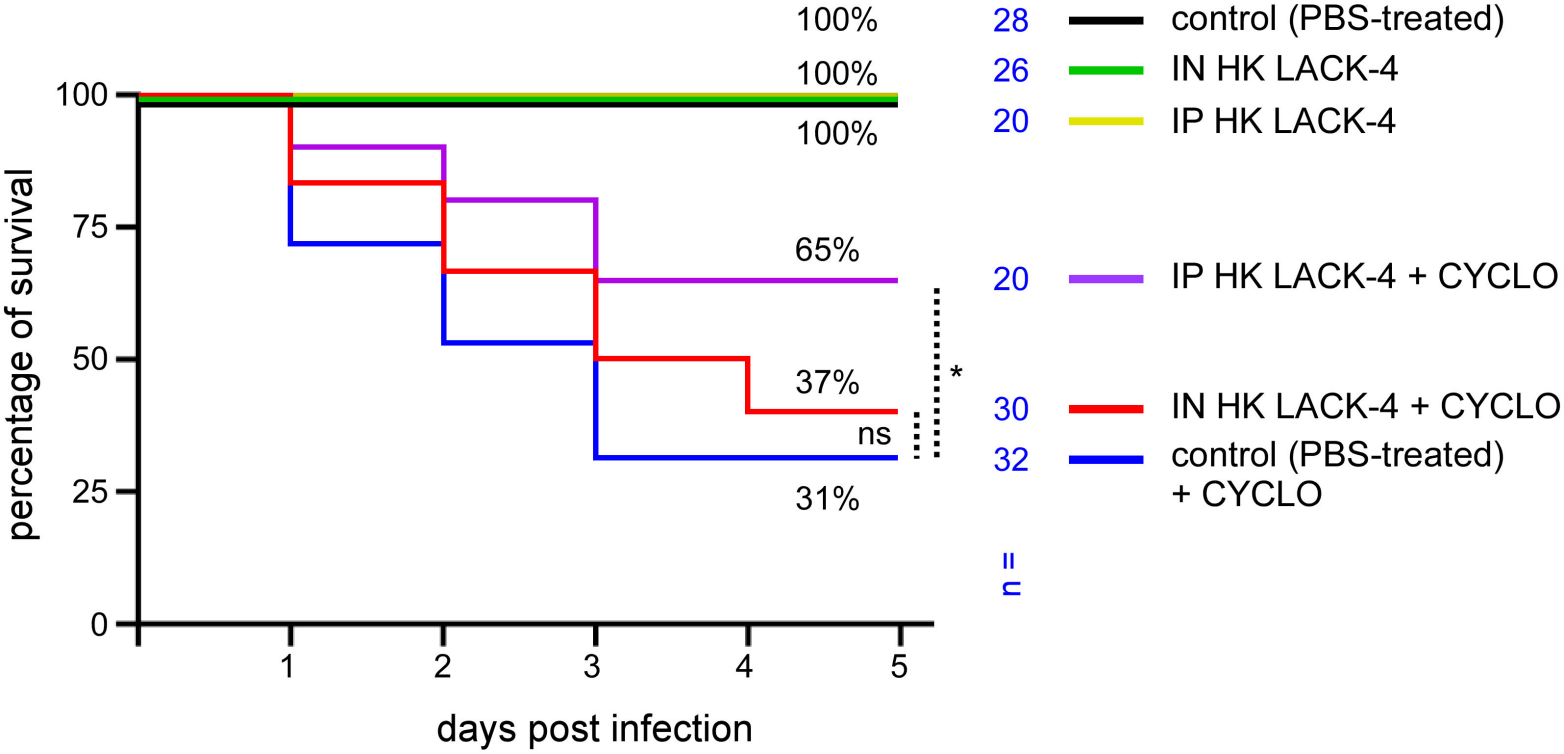
Immunization with HK LAC-4 confers partial protection against LAC-4 challenge to cyclophosphamide-treated wild-type C57BL/6 mice. Wild-type C57BL/6 mice were treated intranasally (IN) with PBS (control) or 10^8^ CFU of heat-killed (HK) LAC-4 *A. baumannii* in PBS. 6 weeks later, control and HK vaccinated mice were challenged intranasally with 2x10^6^ CFU of live LAC-4 strain. Cyclophosphamide (cyclo) was administrated intraperitoneally at 4 days (150 mg/kg) and 1 day (100 mg/kg) before the challenge. Picture shows the percent survival rate. Over the 5 days after challenge, the fitness of the infected mice was monitored. When the human endpoint was reached, the mice were euthanized. These data represent a pool of three independent experiments. n= number of mice for each condition. Significant differences between control (unvaccinated) + CYCLO and vaccinated groups + CYCLO are marked with asterisks: *p < 0.05, in a (Wilcoxon-) Mann-Whitney post-test.

As shown in **Figure 7**, in agreement with a previous study (37), cyclophosphamide treatment greatly reduced the survival of infected wild-type C57BL/6 mice. We observed that intraperitoneal immunization with HK LAC-4 significantly reduced mortality in mice treated with cyclophosphamide. Vaccine protection, however, was not complete in the cyclophosphamide-treated mice and the protection provided did not exceed 65%.

### 2.7 Heat-killed LAC-4 activates human peripheral blood mononuclear cells

To evaluate the ability of HK LAC-4 to induce an adaptive immune response in humans, we measured *in vitro* the activation of dendritic cells and T lymphocytes from Peripheral Blood Mononuclear Cells (PBMC) of healthy donors in response to HK LAC-4.

**Figure 8:**
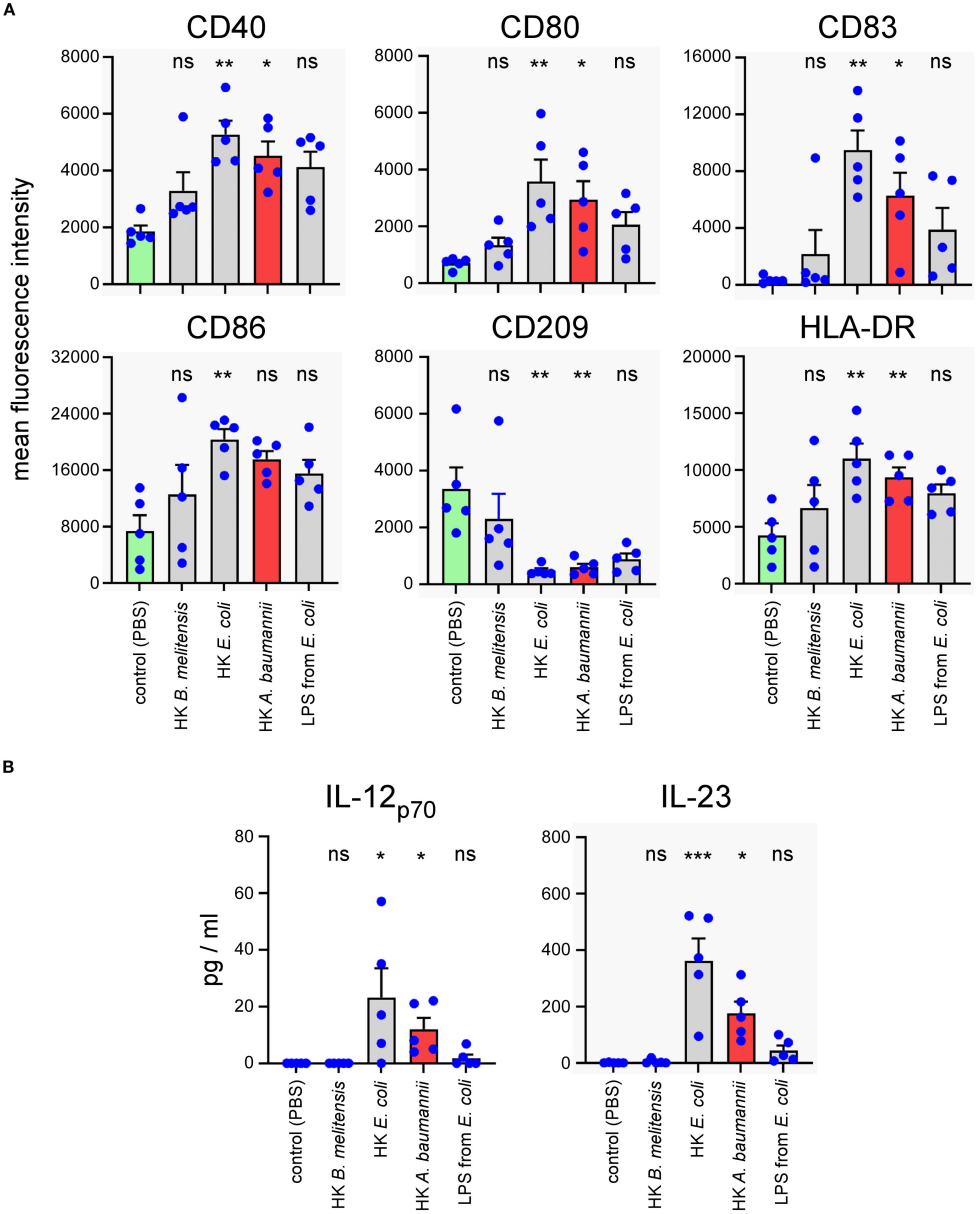
*In vitro* stimulation with heat-killed *A*. *baumannii* LAC-4 induces maturation of human monocyte-derived dendritic cells and the production of IL-23. Monocytes isolated from human PBMC were differentiated for 5 days to dendritic cells with IL-4+GM-CSF and then stimulated with PBS (negative control), heat-killed (HK) LAC-4 *A*. *baumannii*, HK *B*. *melitensis*, HK *E*. *coli* and LPS from *E*. *coli* for 48 hours. **(A)** Six maturation markers were analyzed by flow cytometry and are expressed here as the medium fluorescence intensity (MFI) reflecting the change in expression of the marker on the cell. **(B)** Supernatant from stimulated monocyte-derived dendritic cells was analyzed by ELISA to quantify the cytokines IL-12_p70_ and IL23. Columns represent the mean of five different donors +/- SEM. The stimulated groups are compared to the PBS-treated control group:*p<0.05; **p<0.01; ***p<0.001; determined by the non-parametric 1-way ANOVA with a multiple comparison.

Monocytes isolated from human PBMC were differentiated for 5 days to dendritic cells in the presence of IL-4+GM-CSF and then stimulated for 48 hours with 2x10^6^ CFU/ml of HK *A. baumannii* LAC-4, HK *Brucella melitensis*, HK *Escherichia coli* or 1 µg/ml of LPS from *E. coli*. *B. melitensis* is a stealthy bacterium that expresses an atypical and poorly activating LPS (43). HK *B. melitensis* was used as a negative control in our experiments. We used HK *E. coli* and commercial LPS as positive controls. The expression of dendritic cell surface maturation markers was analyzed by flow cytometry. We observed that HK LAC-4 significantly up-regulated CD40, CD80, CD83 and CD86 maturation markers and down-regulated CD209 (a lectin receptor mostly expressed by immature DCs) (**Figure 8A**). Moreover, HK LAC-4 induced significant secretion of IL-23 and IL-12_p70_ in our experimental setting (**Figure 8B**).

**Figure 9:**
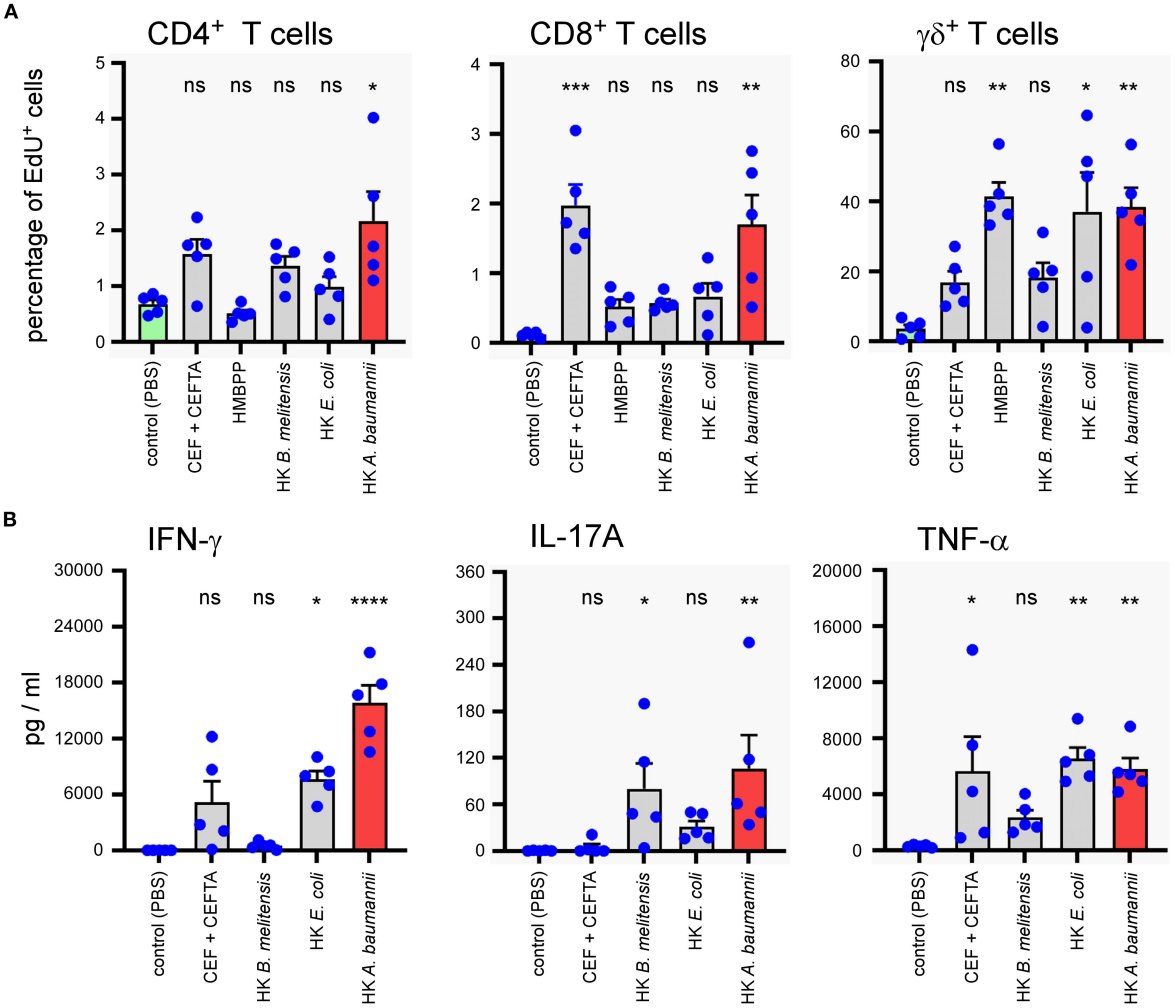
*In vitro* stimulation of human PBMC with heat-killed LAC-4 *A*. *baumannii* induces proliferation of T cells and production of IFN-γ, IL-17A and TNF-α. Total PBMC were stimulated for 7 days with PBS (negative control), CEF+CEFTA (pool of peptides-MHC class I and II-restricted T-cell epitopes) as the positive control for CD4 and CD8 T cell proliferation, HMBPP (4-hydroxy-3-methyl-but-2-enyl pyrophosphate) as the positive control for γδ T cell proliferation, heat-killed (HK) LAC-4 *A*. *baumannii*, HK *B*. *melitensis* and HK *E*. *coli*. **(A)** Proliferation of T cells is expressed by the percentage of cells incorporating EdU (5-ethynyl-2´-deoxyuridine). **(B)** Supernatant from stimulated PBMC was analyzed by ELISA for the quantification of IL-17A and with Luminex for IFN-γ and TNFα. Quantities are expressed in pg/ml. Columns represent the mean of five different donors +/- SEM. The stimulated groups are compared to the PBS-treated control group:*p<0.05; **p<0.01; ***p<0.001; determined by the non-parametric 1-way ANOVA with a multiple comparison.

**Figure 10:**
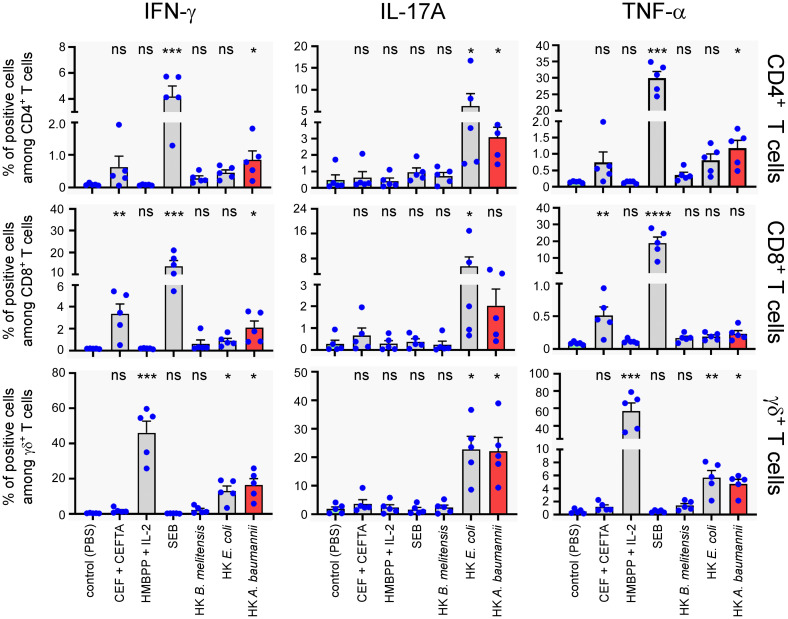
*In vitro* stimulation of human PBMC with heat-killed LAC-4 *A*. *baumannii* of human PBMC induces production of IFN-γ, IL-17A and TNF-α from three distinct T cell populations. Total PBMC were stimulated for 7 days with PBS (negative control), CEF^+^CEFTA (pool of peptides-MHC class I and II-restricted T-cell epitopes), HMBPP (4-hydroxy-3-methyl-but-2-enyl pyrophosphate) + IL-2, SEB, heat-killed (HK) LAC-4 *A*. *baumannii*, HK *B*. *melitensis* or HK *E*. *coli*. Brefeldin-A and Monensin were added for the last 5 hours before intracellular FACS staining. For stimulation with HMBPP + IL-2, the compounds were added for 5 hours in culture media treated cells, at the time of Brefeldin-A and Monensin blocking. Graphs represent the percentages of cells expressing IFN-γ, IL-17A or TNF-α in the three main T cell populations. Columns represent the mean of five different donors +/- SEM. The stimulated groups are compared to the PBS-treated control group: *p<0.05; **p<0.01; ***p<0.001; ****p<0.0001; ****p<0.0001 determined by the non-parametric 1-way ANOVA with a multiple comparison.

To evaluate the capacity of HK LAC-4 to activate human T lymphocytes, total PBMC were stimulated for 7 days with 2x10^6^ CFU/ml of HK *A. baumannii* LAC-4, HK *B. melitensis* or HK *E. coli*. We used 2 µg/ml CEF+CEFTA (pool of peptides-MHC class I and II-restricted T-cell epitopes from human Cytomegalovirus, Epstein Barr virus, Influenza virus, Tetanus toxin and Adenovirus 5) and 1 µg/ml of SEB (Staphylococcal enterotoxin B) as the positive control for CD4^+^ and CD8^+^ T cell proliferation and 10 µg/ml HMBPP (4-hydroxy-3-methyl-but-2-enyl pyrophosphate) as the positive control for γδ^+^ T cell proliferation. We observed that HK LAC-4 induced significant proliferation of CD4^+^ T cells, CD8^+^ T cells and γδ+ T cells (**Figure 9A**) as well as easily detectable secretion of IFN-γ, IL-17A and TNF-α (**Figure 9B**). To determine the cell source of these cytokines, we analyzed stimulated total PBMC by intracellular flow cytometry. Our results showed (**Figure 10**) that HK LAC-4 induced significant production of IFN-γ by CD8^+^ and γδ+ T cells, of IL-17A by CD4^+^, CD8^+^ and γδ ^+^ T cells and of TNF-α by CD4^+^ and γδ+ T cells.

Overall, these results show that HK LAC-4 can induce *in vitro* dendritic cell maturation as well as activation of all T cell subsets, suggesting that it might be able to activate lymphocytes in patients.

## 3 Discussion

Given its growing significance as a public health threat, considerable efforts have been directed toward developing vaccines against *A. baumannii*. Numerous vaccine candidates have been evaluated in animal models, such as subunit (44, 45), DNA, Outer membrane vesicles, inactivated whole-cell and live attenuated vaccines (46). However, no vaccine candidates have advanced into clinical trials.

*A. baumannii* displays marked genetic and phenotypic diversity (8, 11) and causes opportunistic infections in individuals, some of whom experience varying degrees of disease- or treatment-related immunosuppression (3). These factors make it unlikely that subunit or live-attenuated vaccines would achieve widespread success in real-world settings. The plasticity of the *A. baumannii* genome means that an attenuated vaccine could recover its virulence and thus be fatal to immunocompromised individuals. This plasticity could also allow it to quickly escape the immunity conferred by a subunit vaccine which would be focused on a small number of antigenic determinants. Consequently, a whole inactivated vaccine could represent a promising solution by avoiding any risk of reversion of its virulence and providing immunization against many *A. baumannii* antigens.

Several whole inactivated vaccines, such as formalin-killed ATCC 19606 strain (47), formalin-killed LAC-4 strain (33) or γ-irradiated (48) and ultraviolet C–inactivated (49) AB5075 strain, have been shown to protect mice against intraperitoneal or intranasal *A. baumannii* infection. In this study, we test a vaccine composed of heat-inactivated LAC-4 bacteria. We chose this protocol of inactivation for its practicality, safety, and simplicity, so that it is easy to reproduce by all experimenters. Our choice was also informed by previous reports documenting that formalin treatment can alter protein presentation to T cells (50) and affect the immunogenicity of some vaccines, such as vaccine against pertussis toxin (51), poliovirus (52) and *Pseudomonas aeruginosa* and *Staphylococcus aureus* (53). Moreover, it has been showed in some models that heat-inactivated vaccines may elicit stronger immune responses than their formalin-inactivated counterparts (54).

**Figure 11:**
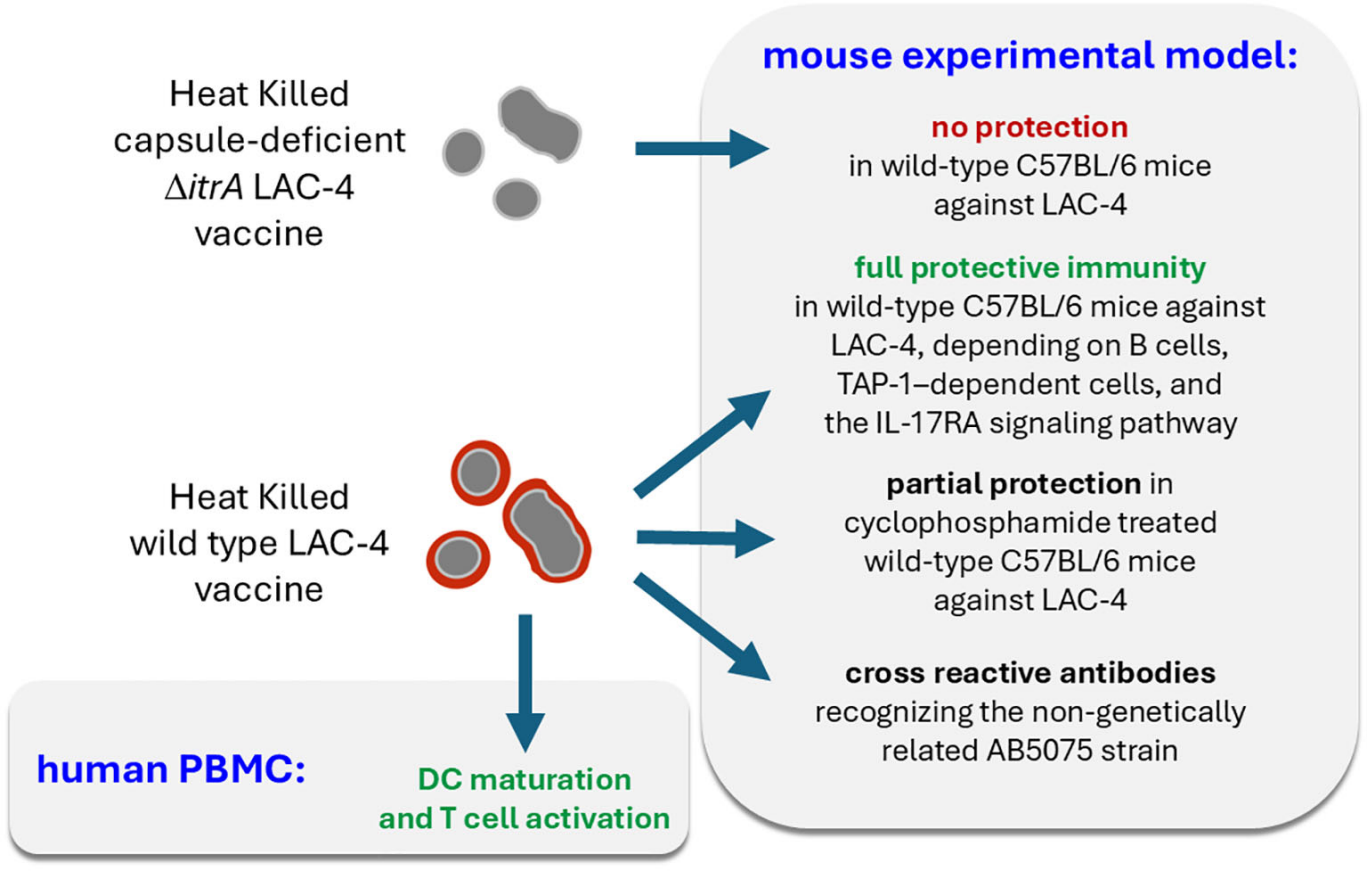
Schematic representation of the main results.

In the present work, using heat-killed (HK) LAC-4 vaccine, we confirmed previous findings (35) demonstrating the protective efficacy of a formalin-inactivated LAC-4 strain against lethal lung infection with the hypervirulent LAC-4 strain in wild-type C57BL/6 mice. This protection was absent when using a vaccine prepared from the capsule-deficient LAC-4 Δ*itrA* strain, suggesting that the immune response targets mainly non-protein surface antigens. We observed that a HK vaccine composed of the AB5075 strain, which is genetically unrelated to LAC-4, could induce antibodies capable of recognizing LAC-4 antigens and a partial protection against LAC-4 infection. We also observed that the protection induced by the HK LAC-4 vaccine was critically dependent on B lymphocytes. Then, we further showed that a single dose of the LAC-4 vaccine can partially protect certain genetically immunodeficient mouse models susceptible to *A. baumannii* pulmonary infection, including IL-17RA^-/-^, γTCR^-/-^, TAP-1^-/-^, and CD3^-/-^ mice, as well as wild-type mice rendered immunocompromised by cyclophosphamide treatment—an immunosuppressive drug widely used in patients with cancer or autoimmune diseases (36). Moreover, we demonstrated that the HK LAC-4 vaccine can activate human dendritic cells and T lymphocytes *in vitro*, suggesting its potential to stimulate adaptive immunity in patients. All of these results are summarized schematically in **Figure 11**.

The precise mechanisms explaining the protective effect of the HK LAC-4 vaccine remain to be elucidated. Our vaccine fails to completely protect MuMT^-/-^, IL-17RA^-/-^, γTCR^-/-^, and TAP-1^-/-^ mice against a high dose of LAC-4, suggesting that the IL-17RA-dependent signaling pathway and IgM, γTCR- and TAP-1-dependent cell populations are essential for optimal protection against *A. baumannii*.

The humoral response appears to be a key element of protection induced by the HK LAC-4 vaccine since MuMT deficiency in B cells completely abrogates protection in vaccinated mice. We have shown that HK LAC-4 vaccine induces IgM, IgG1 and IgG3 antibodies recognizing mainly the non-proteinaceous capsule antigens of the LAC-4 strain. A recent study (55) reported that a monoclonal antibody targeting cell-surface pseudaminic acid of *A. baumannii* exhibits direct bactericidal activity, supporting the possibility that antibodies directed against the capsule may confer protection. Interestingly, we have observed that antibodies induced by HK AB5075 not only recognize AB5075 but also cross-react with LAC-4. This cross-reaction confers significant protection as demonstrated by increased survival and reduced bacterial counts in the lungs and spleen of mice vaccinated with HK AB5075 and challenged with live LAC-4 compared to unvaccinated mice. These results suggests that a whole HK vaccine comprising a large panel of antigens could potentially confer protection against several different strains of *A. baumannii*. A promising vaccination strategy would be to create a multivalent inactivated vaccine combining several field strains among those most involved in human infections to ensure the broadest possible protection. However, the feasibility of this approach requires better identification of surface antigens that are shared between different strains and those that are not. Surface omics approaches (56), analyzing both proteins, sugars, and lipids exposed on the surface of *A. baumannii* strains, could help select the most interesting strains to combine in vaccines.

We attempted to protect naive wild-type mice using serum transfer from vaccinated mice. These transfers resulted in weak, statistically insignificant, and poorly reproducible protection in preliminary experiments. Therefore, we did not explore this path further. These results are similar to that obtained by KuoLee et al. (35), who demonstrated that the transfer of immune serum only prolongs survival by approximately 48 hours after infection with the LAC-4 strain. The difficulty in transferring protection via serum suggests that cellular components participate directly in protection. We observed that the cellular response induced by HK LAC-4 in lungs and spleen seems mainly directed towards the production of Th17 cytokines, such as IL-17A, IL-17F and IL-22, which are particularly important for inducing the production of antimicrobial peptides by the pulmonary mucosa (57).

Comparing the survival rates of different strains of mice genetically deficient for key elements of the adaptive response to LAC-4 infection opens up interesting new avenues of research. We observed that deficiencies in IL-17RA, δTCR and TAP-1 strongly reduce the survival of naïve mice and are associated with a loss of control of the multiplication of *A. baumannii* in the lungs, which has not been described previously to our knowledge. However, these results should be interpreted with caution. TAP-1 is involved in the association of cytosolic antigens with MHC-I and the absence of this molecule has been shown to prevent the development of CD8^+^ T cells (58). But its absence has also been found to have an effect on natural killer T cells (59) and on natural killers cells (60). Depletion of CD8^+^ T cells in C57BL/6 mice by administration of monoclonal antibody did not induce an increase of susceptibility to intranasal infection by *A. baumannii* (26). In contrast, depletion of NK1.1^+^ cells (a marker shared by NK and some NKT cells) in C57BL/6 mice increases mortality and reduces neutrophil recruitment during *A. baumannii* infection (61), suggesting that these cell types could participate in early defense against *A. baumannii* infection. IL-17RA recognizes IL-17A, IL-17E and IL-17F and has been implicated in the control of extracellular pathogens at the mucosal level (62), which could explain the high susceptibility of IL-17RA^-/-^ mice observed in our infection model. As γδ T cells are known to produce IL-17A (63), we could hypothesize that a deficiency in γδ T cells results in lower production of IL-17A in mice infected with LAC-4. However, our data show that δTCR^-/-^ mice produce as much IL-17A, IL-17F, IL-22 and TNF-α as wild-type mice. Furthermore, IL-17^-/-^ C7BL/6 mice have been reported to be resistant to pulmonary LAC-4 infection (64). Thus, further investigation will be required to formulate definitive hypotheses explaining the susceptibility of IL-17RA signaling in the control of *A. baumannii* infection. This constitutes a priority for future research.

Results demonstrating partial protection in animals genetically deficient for key components of the immune response or animals treated with cyclophosphamide are particularly encouraging as immunocompromised patients are frequently infected by *A. baumannii* (9) and constitute an at-risk population during all epidemics. These populations not only suffer the most serious infections but are also poorly receptive to most vaccines and can act as reservoirs for pathogens, providing them opportunities to adapt to the host immune defense and to antibiotic treatments. For example, it is well known that immunocompromised patients are less well protected against SARS-CoV-2 by vaccines (65, 66). In addition, SARS-CoV-2 variants able to escape antiviral drugs (67) and adaptive immunity (68–70) are suspected to have emerged following chronic infection of immunocompromised individuals. This escape phenomenon is not limited to SARS-CoV-2, and has been documented with other viruses (71, 72) as well as with bacteria (73). It is therefore particularly important, both in the interest of patients and the wider population, to successfully develop vaccines that effectively protect immunocompromised individuals. Our study demonstrates that this is possible in the experimental model of pulmonary infection with *A. baumannii*.

A previous study by Dolery et al. (48) reported stronger protection than we observed in mice vaccinated with a gamma-irradiated *A. baumannii* strain, treated with cyclophosphamide, and subsequently infected intranasally with *A. baumannii*. Several differences between their model and ours likely account for this discrepancy. First, Dolery et al. used the AB5075 challenge strain, which is less virulent than the LAC-4 strain used in our experiments (see **Supplementary Figure S1**). Second, our vaccination protocol consisted of a single dose, whereas theirs involved three administrations. Third, in their study, the challenge occurred only two weeks after the final boost, compared with six weeks after vaccination in our model. Intranasal administration of inactivated LAC-4 has been shown to induce an innate immunity–mediated protective phase lasting at least seven days post-administration (34). Therefore, the stronger protection reported by Dolery et al. may result from the combination of multiple boosts, the short interval between the last boost and the challenge, and the lower virulence of the AB5075 strain.

Finally, as experimental mouse models often fail to predict the immunogenicity of a vaccine in humans, we analyzed *in vitro* the ability of HK LAC-4 to induce the activation of human monocyte-derived dendritic cells (DCs) and T cells. We found that HK LAC-4 is a potent activator, capable of inducing the expression of co-stimulatory signals on DCs and of inducing T cell proliferation and cytokine production, suggesting that HK LAC-4 could potentially activate these components of adaptive immune system of patients and induce the development of a protective memory response against *A. baumannii*.

Despite our promising findings, our study nevertheless has several important limitations. *A. baumannii* is known to exhibit enormous genetic and phenotypic variation. Our study focused on the LAC-4 strain which is described as hypervirulent in mice. Our results would be further substantiated if similar results were obtained with other strains of *A. baumannii*. In this study, we showed that HK LAC-4 is a very good activator of the murine and human immune systems. This is encouraging for the development of a vaccine but should elicit caution to its possible side effects. HK LAC-4 would likely be too proinflammatory to be used safely in humans. Strategies to enhance antigen exposure while minimizing inflammation include reducing the antigen dose, splitting doses (prime and boost), choosing a safer administration route (e.g., subcutaneous injection), and using a matrix for controlled antigen release. A recent study by Luzuriaga et al. (74) demonstrated that it was possible to inactivate bacteria and make them more immunogenic by biomimetically mineralizing them within a metal−organic framework. This process makes it possible to increase specific antibody levels induced as well as the level of protection against an extracellular bacterium such as the *E. coli* CFT073 strain responsible for urinary tract infections.

Taken together, our results improve our understanding of the protective mechanisms involved in the immune response against *A. baumannii*. They also suggest that the development of vaccines capable of protecting immunocompromised patients against opportunistic infections such as those caused by *A. baumannii* is possible.

## 4 Materials and methods

### 4.1 Ethics statement

The procedures used in this study and the handling of the mice complied with current European legislation (Directive 86/609/EEC). The Animal Welfare Committee of the Université de Namur (UNamur, Belgium) reviewed and approved the complete protocol for *Acinetobacter baumannii* infection (Permit Number: UN-LE-20/343, UN-LE-22/378). Whole blood for the isolation of human PBMC was collected from healthy donors as described in the ethical protocol/amendment IXP-001_V3 (Belgium; Reg. No. B6702014215858), protocol IXP-003_V1 (Belgium; Reg. No. B707201627607) or protocol IXP-004_V1 (The Netherlands; Reg. No. NL57912.075.16).

### 4.2 Mice, PBMC and bacterial strains

Wild-type C57BL/6 mice were obtained from Harlan (Bicester, UK). IFN-γ^−/−^, δTCR^−/−^, CD3ϵ^−/−^, MuMT^−/−^ C57BL/6 mice were purchased from The Jackson Laboratory (Bar Harbor, ME). IL-12_p35_^−/−^ C57BL/6 mice were obtained from Dr. B. Ryffel (University of Orleans, France). IL-17RA^−/−^ C57BL/6 mice were obtained from Dr. K. Huygen (Belgian Scientific Institute for Public Health, Brussels, Belgium). TAP1^−/−^ C57BL/6 mice and MHCII^−/−^ C57BL/6 mice were acquired from Jörg Reimann (University of Ulm, Ulm, Germany). All wild-type and deficient mice used in this study were bred in the animal facility of the Gosselies campus of the Université Libre de Bruxelles (ULB, Belgium).

Cryopreserved primary PBMC were isolated from whole blood donated by healthy volunteers. Written informed consent was obtained from all patients enrolled in the study. All blood samples were tested and found negative for hepatitis B virus (HBV), hepatitis C virus (HCV), and human immunodeficiency virus (HIV). PBMC were separated from the blood by density gradient centrifugation and subsequently cryopreserved in fetal bovine serum (FBS), supplemented with 10% dimethyl sulfoxide, by controlled rate freezing. The PBMC were kept in cryogenic storage (−180 °C) until use.

*A. baumannii* LAC-4 (31, 32) (ASM78673v1 genome, https://www.ncbi.nlm.nih.gov/datasets/genome/GCF_000786735.1/) was obtained from Wangxue Chen (Human Health Therapeutics Research Center, National Research Council of Canada, Ottawa, ON, Canada). *A. baumannii* AB5075 was isolated in 2008 from a combatant wound infection (38) and was obtained from Dr. C. Van der Henst (Structural Biology Brussels, Vrije Universiteit Brussel (VUB) and Microbial Resistance and Drug Discovery, VIB-VUB Center for Structural Biology, VIB, Flanders Institute for Biotechnology, Brussels, Belgium). *A. baumannii* was always handled in BSL-2 or BSL-3 containment at the Université Libre de Namur (UNamur, Namur).

### 4.3 Construction of the itrA gene deletion mutant

To generate the non-capsulated *A. baumannii* LAC-4 strain, the itrA3 gene (ABLAC_36890 from the LAC-4 reference genome CP007712) was obtained using the integrative plasmid pK18-apraR [pUC derivative (75)] generated using the following primers: itrA3_up_for_short: GCAAGTATCGAAGCAATATCAG; itrA3_up_rev_short:GGCCCAATTCGCCCTATAGTGAGTCGGGATTAATGCGGTTAAGGAAATTACG; itrA3_down_rev_short: CCTAATTTTGGATGTTCTAAGCC; itrA3_down_for_short:CCGACTCACTATAGGGCGAATTGGGCCGCTCCAGATCAGTTAGTAGG. Briefly, the pK18-Δ*itrA3* was integrated into the parental wild-type LAC-4 strain and excised using the sucrose counter-selection marker as previously described (42). The lack of capsule of the LAC-4ΔitrA3 strain was confirmed by density gradients as previously described (76).

### 4.4 *A. baumannii* infection

Bacteria stored at -80°C in LB-glycerol 30% were inoculated in LB medium and incubated overnight at 37°C under agitation until the stationary phase. Then the culture was diluted to an OD600 (Optical Density measured at a wavelength of 600 nm) of 0.2 for AB5075 and 0.4 for LAC-4 and then was grown until an OD600 of 0.7-0.8. This log-phase culture was then centrifugated for 10 minutes at 5000 g and the pellet was washed twice with Phosphate-Buffered Saline (PBS). Then it was diluted to the target concentration prior to infection. The infectious dose was assessed by plating serial dilutions.

Mice were anesthetized with a cocktail of Xylazine (9 mg/kg) and Ketamine (36 mg/kg) in PBS before being inoculated by intranasal injection with the indicated dose of *A. baumannii* in 30 µl of PBS. Mice infected intraperitoneally were injected with the indicated dose in 500 µL. Control animals were inoculated with the same volume of PBS. The infectious doses were validated by plating serial dilutions of the inoculums. At the selected time after infection, mice were sacrificed by cervical dislocation. Immediately after sacrifice, the lungs and spleen were collected for bacterial count and qRT-PCR analyses.

### 4.5 Preparation of heat-killed bacteria

To carry out vaccinations as well as *in vitro* tests, different species and strains of bacteria were killed by heat. We used the previously described *A. baumannii* strains as well as *Escherichia coli* K12 MG1655 and *B. melitensis* strain 16M (Biotype1; ATCC 23456; American Type Culture Collection). To prepare Heat-Killed (HK) bacteria, overnight cultures of bacteria were grown under shaking at 37°C in 2YT media (Luria-Bertani (LB) broth 32 g/L with yeast extract 5 g/L and peptone 10 g/L) for *B. melitensis* and in LB media for *A. baumannii* and *E. coli*. *B. melitensis* and *E. coli* from an overnight liquid culture were washed twice in PBS (10 minutes at 3500 g for *Brucella* and 5000 g for *E. coli*). *A. baumannii* from an overnight liquid culture were washed twice in PBS (10 minutes at 5000 g) and diluted to an optical density of 0.4 in fresh LM media and growth for 90 minutes at 37°C under constant agitation. and then washed twice in PBS. After washing, the bacteria were resuspended to a concentration of 2x10^9^ CFU/ml in PBS. This solution was heated at 80°C for 1 hour in a water bath. Following this treatment, the bacterial solution was stored at -20°C before use. Each batch of killed bacteria was verified to confirm the killing by growing it in an incubator at 37°C and counting the CFU after 24 hours for *A. baumannii* and *E. coli* and after 72 hours for *B. melitensis*.

### 4.6 Vaccination

C57BL/6 mice were unvaccinated or vaccinated by intranasal or intraperitoneal injection with 10^8^ CFU of strains of the *A. baumannii* of interest, as indicated. Samples of blood for antibody detection were collected 4 weeks post-vaccination for analysis of the humoral response against *A. baumannii*. Six weeks post-vaccination, mice were challenged intranasally with live LAC-4 bacteria (2x10^6^ or 2x10^7^ CFU, as indicated). In some experiments, monohydrate cyclophosphamide (Sigma) diluted in PBS was administrated intraperitoneally at 4 days (150 mg/kg) and 1 day (100 mg/kg) before the challenge. Lungs and spleen were harvested 24 and 120 hours post-challenge and the CFU levels in each organ were counted.

### 4.7 Bacterial counting

Organs were homogenized in PBS/0.1% X-100 Triton (Sigma-Aldrich). We performed successive serial dilutions in PBS to obtain the most accurate bacterial count and plated them on LB medium. The CFU were counted after 1 day of incubation at 37°C.

### 4.8 RNA extraction from lungs and spleen

RNA from the whole lungs and spleen was extracted by the TRIzol™/Chloroform method. Briefly, the lungs and spleen were harvested from mice and ground in 1 mL of TRIzol™ (TRIzol™ Reagent - Invitrogen™) with a Polytron Homogenizer. After 5 minutes of incubation at room temperature, 200 µL of chloroform were added and the tube was mixed vigorously, then incubated for 2–3 minutes at room temperature. After centrifugation for 15 minutes (12,000 g at 4°C), the aqueous phase was recovered and RNA was extracted using the “NucleoSpin RNA Plus (Macherey-Nagel)” kit according to the manufacturer’s protocol.

### 4.9 qRT-PCR analysis of cytokines

The RNA was then reverse transcribed with Superscript II reverse transcriptase (Invitrogen) using hexamer random primers as described by the manufacturer. A condition without reverse transcriptase was also conducted in parallel as a negative control. cDNA was then mixed with SybrGreen mix (FastStart Universal SYBR Green Master (Rox) - Roche) and the appropriate primer sets and subjected to qRT-PCR in a LightCycler 480 (Roche). The forward and reverse primers used were 5’-GGCAACTGTTCCTGAACTCA-3’ and 5’-GGGTCCGTCAACTTCAAAGA-3’ for IL-1β, 5’-CCTGTCTATACCACTTCACA-3’ and 5’-CTCTTTTCTCATTTCCACGATTTCC-3’ for IL-6, 5’-GCCTCCCTCTCATCAGTTCTA-3’ and 5’-GCTACGACGTGGGCTACAG-3’ for *TNF-*α, 5’-TGCCAAGTTTGAGGTCAACA-3’ and 5’-GAATCAGCAGCGACTCCTTT-3’ for *IFN-γ*, 5’-ATCCCTCAAAGCTCAGCGTGTC-3’ and 5’-GGGTCTTCATTGCGGTGGAGAG-3’ for *IL-17A*, 5’-CCCTGGAGGATAACACTGTGA-3’ and 5’-AATTCACGTGGGACAGAAATG-3’ for *IL-17F* and 5’-CAGCAGCCATACATCGTCAA-3’ and 5’-GCCGGACATCTGTGTTGTTA-3’ for *IL-22*. Ribosomal Protein L32 (*RPL32*, forward 5’-ACATCGGTTATGGGAGCAAC-3’, reverse 5’-TCCAGCTCCTTGACATTGTG-3’) mRNA was used as the reference housekeeping gene for normalization. A total of 40 two-step cycles were performed as follows: 95°C for 15 seconds and 60°C for 1 minute preceded by 10 minutes at 95°C for activation. Melting curves were then performed to assess the primer specificity. The target mRNA fold change was calculated based on the 2^-ΔΔCt^ formula, using the *RPL32* gene as the reference gene and RNA from naïve mice as the standard condition. Four biological replicates were performed for each gene tested.

### 4.10 Enzyme-linked immunosorbent assay to detect anti-*A. baumannii* antibodies

The presence of *Acinetobacter baumannii*-specific murine IgM, IgG1 and IgG3 was determined by ELISA. Polystyrene plates (269620; Nunc) were coated with Heat-Killed (HK) *A. baumannii* LAC-4 or AB5075 at a dose of 10^7^ CFU/mL, as indicated, and incubated overnight at 4°C. The plates were blocked for 2 hours at room temperature with 200 μl/well of PBS-1% Bovine Serum Albumin (BSA). Then, plates were incubated with 50 μl/well of plasma in serial dilutions in PBS-0.1% BSA. The plasma of uninfected mice and PBS were used as negative controls. After four washes with PBS, isotype-specific goat anti-mouse HRP conjugated antibodies were added (50 μl/well) at appropriate dilutions (anti-IgM from Sigma-Aldrich; anti IgG1 LO-MG1–13 HRPO and LO-anti IgG3 MG3–13 HRPO from LOIMEX). After 1 hour of incubation at room temperature, plates were washed four times in PBS and 100 μl/well of TMB substrate solution (BD OptEiA Kit) was added. After 15 minutes of incubation at room temperature in the dark, the enzyme reaction was stopped by adding 25 μl/well of 2 N H_2_SO_4_. The absorbance was measured at 450 nm.

### 4.11 Analysis of antigen profiles by Western blot

Bacterial cultures of *A. baumannii* were normalized at 2 x 10^9^ bacteria/mL, then heat killed as previously described. Two milliliters of culture were concentrated in 300 µL of PBS containing the loading buffer (2% SDS, 5% β-mercaptoethanol, 10% glycerol, 0.03 M Tris HCl pH6.8) and samples were heated at 90 °C for 10 minutes before being loaded on 4-20% mini-PROTEAN acrylamide gels (BioRad), with 20 µL of samples per well. Migration was performed for 1 hour at 150V. After migration, one gel was kept for Coomassie Blue staining, while the others had their proteins transferred onto a nitrocellulose membrane which was blocked overnight in PBS supplemented with 0.05% Tween (PBS-T) and 5% milk. For primary antibodies, sera from 8 different vaccinated mice were pooled together and diluted 4 times in PBS-T supplemented with 1% milk. Incubation of the membranes with their respective primary antibodies were performed statically for 1 hour on clean petri dishes with drops of 300 µL for each membrane. Membranes were then washed in PBS-T 4 times for 5 min, before being incubated with secondary antibodies (polyclonal Rabbit anti-Mouse Ig/HRP, Agilent) diluted 5000 times in PBS-T supplemented with 1% milk. After 4 washings, proteins were revealed using Clarity Western ECL Substrate (Biorad) with Image Quant LAS 4000 (General Electric).

### 4.12 Human dendritic cell maturation assay

Peripheral Blood Mononuclear Cells (PBMC) from healthy human donors were thawed from the ImmunXperts biobank. Monocytes were isolated from PBMC using a MACS magnetic separation column (Miltenyi) following the manufacturer’s instructions and purity was evaluated by CD14 staining using a BD Lsrfortessa X-20 flow cytometer. Cells were then resuspended to a cellular density of 10^6^cells/ml and plated on a tissue culture treated 24-well plate (1ml per well) in CellGenix DC medium (CellGenix) with added Gentamicin antibiotic (Sigma-Aldrich), IL-4 and GM-CSF for 5 days. At day 5, cells were stained for FACS analysis with several DC activation markers to assess their immature (iDC) state (CD14 Miltenyi, CD40 BD, CD80 BD, CD83 Miltenyi, CD86 Miltenyi, CD209 Miltenyi and HLA-DR BD). On the same day, the appropriate stimuli (various heat-killed bacteria and LPS from *E. coli* from Sigma-Aldrich) were added to the cell culture for 48 hours. At day 7, cells were stained for FACS analysis with the same markers used at day 5 to assess their mature state (mDC). Supernatant at day 7 was collected for further analysis.

### 4.13 ELISA to detect human cytokines

Supernatant from the cell culture was collected and stored at -80°C. After thawing, the presence of the cytokines IL-12p70, IL-23 and IL-17A was detected using LEGEND MAX™ Human ELISA kits (BioLegend) with pre-coated plates following the manufacturer’s instructions.

### 4.14 Luminex to detect human cytokines

Supernatant from cell culture was collected and stored at -80°C. After thawing, the presence of the cytokines IFN-γ and TNF-α was detected using the Luminex technology following the manufacturer’s instructions (MILLIPLEX MAP Human Cytokine/Chemokine kit, Merck).

### 4.15 Proliferation assay on human PBMC

Peripheral Blood Mononuclear Cells (PBMC) from healthy human donors were thawed from the ImmunXperts biobank and incubated for 6 days at 37°C with the appropriate stimuli: CEF+CEFTA (pool of 35 peptides-MHC class I and II-restricted T-cell epitopes from human Cytomegalovirus, Epstein Barr virus, Influenza virus, Tetanus toxin and Adenovirus 5 from Mabtech), HMBPP (4-Hydroxy-3-methylbut-2-enyl diphosphate lithium salt, Sigma-Aldrich) and various heat-killed bacteria. T cell proliferation was assessed by measuring the incorporation of 5-Ethynyl-2´-deoxyuridine (EdU). EdU is a thymidine analogue which is incorporated into the DNA of dividing cells during the S phase. On day 6, cell cultures were pulsed with EdU at 1 µM for approximately 16 hours. The next day, cell culture supernatants were collected and stored at -80°C for cytokine analysis. The remaining cells were fluorescently stained for viability and T cell surface markers (CD3 BD, CD4 BD, CD8 BD and γδTCR-Miltenyi), fixed, permeabilized and the incorporated EdU was stained with a fluorescent azide (Click-iT™ EdU Flow Cytometry Assay Kit, Invitrogen, Thermofisher Scientific). Cells were then acquired on a BD Lsrfortessa X-20.

### 4.16 Intracellular cytokine staining on human PBMC

Peripheral Blood Mononuclear Cells (PBMC) from healthy human donors were thawed from the ImmunXperts biobank and incubated for 7 days at 37°C with the appropriate stimuli. At day 7, part of the supernatant was collected, and the remaining cells were treated with Brefeldin A/Monensin (Sigma) for 4–5 hours at 37°C following the manufacturer’s instructions. For some conditions, cells were only stimulated for the last 4–5 hours with Brefeldin A/Monensin. Cells were then stained for viability and extracellular markers (CD3-BD, CD4-BD, CD8-BD and γδTCR-Miltenyi), fixed and permeabilized (BD Cytofix/Cytoperm™ Fixation/Permeabilization Kit) and intracellularly stained for the cytokines IFN-γ-BD, TNF-α-BD and IL-17A-eBiosciences. Cells were then acquired on a BD Fortessa X-20.

### 4.17 Statistical analysis

For comparisons of CFU counts between groups, statistical analyses were performed using the non-parametric one-way ANOVA followed by multiple comparison tests in GraphPad Prism. Survival curves were compared using the log-rank (Mantel–Cox) test in GraphPad Prism. To analyze ELISA data, we use two-way Anova with Dunnett’s multiple comparisons test in GraphPad Prism. A p-value < 0.05 was considered statistically significant (*p < 0.05, **p < 0.01, ***p < 0.001, ****p < 0.0001).

## Data Availability

The raw data supporting the conclusions of this article will be made available by the authors, without undue reservation.

## Supplementary material

Supplementary Figure 1 Comparison of the virulence of LAC-4 and AB5075 strains in the C57BL/6 mouse intranasal infection model. Wild-type C57BL/6 mice were inoculated intranasally (IN) with 2x10^6^ or 2x10^6^ CFU of LAC-4 or HK 5075 A*. baumannii* strains. **(A)** At 4, 24 and 48 hours post infection, some mice were sacrificed and the number of bacteria per gram in the lungs and spleen was evaluated by CFU counting. Red lines represent the geometric mean for each group of mice. These data are representative of two independent experiments. **(B)** During the 120 hours following infection, the fitness of infected mice was monitored. When the human endpoint was reached, the mice were euthanized. These data represent a pool of two independent experiments. n = number of mice per group. Significant differences between groups are marked with asterisks: *p < 0.05, ***p < 0.001, ****p < 0.0001, in a log-rank (Mantel–Cox) test for survival curve and a (Wilcoxon-) Mann-Whitney post-test for CFU count.

Supplementary Figure 2 Production of IL-17A, IL-17F, IL-22, and TNF-α is not reduced in δTCR-deficient mice in response to infection with *A. baumannii* LAC-4 strain. Wild-type and δTCR^-/-^ C57BL/6 mice were inoculated intranasally with PBS (control) or 2x10^6^ CFU of live *A. baumannii* LAC-4 strain in PBS, as indicated. At 4 and 24 hours post-infection, mice were sacrificed, and lungs were collected. Expression levels of the indicated genes were assessed by quantitative RT-PCR and expressed as relative expression to RPL32 housekeeping mRNA (log_10_). The naïve mice (number 1) condition was set to 1. n = 4 mice for each condition. Significant differences between groups (B) are marked with asterisks: ****p < 0.0001, in a (Wilcoxon-) Mann-Whitney post-test.

Supplementary Figure 3 Evaluation of *A. baumannii* LAC-4 infection control in different stains of C57BL/6 mice. Wild-type and immunodeficient C57BL/6 mice were infected intranasally with LAC-4 *A. baumannii* (2.10^6^ CFU) and sacrificed at 24 hours post-infection. The data represent the number of CFU/g of lungs or spleen, as indicated. Red lines represent the geometric mean. Dotted red line represents the mean of the results for wild-type mice. Significant differences between wild-type and deficient mice are marked with asterisks: *p < 0.1, **p < 0.01, ***p < 0.001, ****p < 0.0001, in a (Wilcoxon-) Mann-Whitney post-test.
