# Supplementary figures and images for: Humoral response induced after intranasal vaccination with heat inactivated Acinetobacter baumannii protects immunodeficient mice against hypervirulent LAC-4 strain
Source: Front Immunol. 2025 Sep 22;16:1641997. doi: 10.3389/fimmu.2025.1641997 (PMC12498113; doi:10.3389/fimmu.2025.1641997)

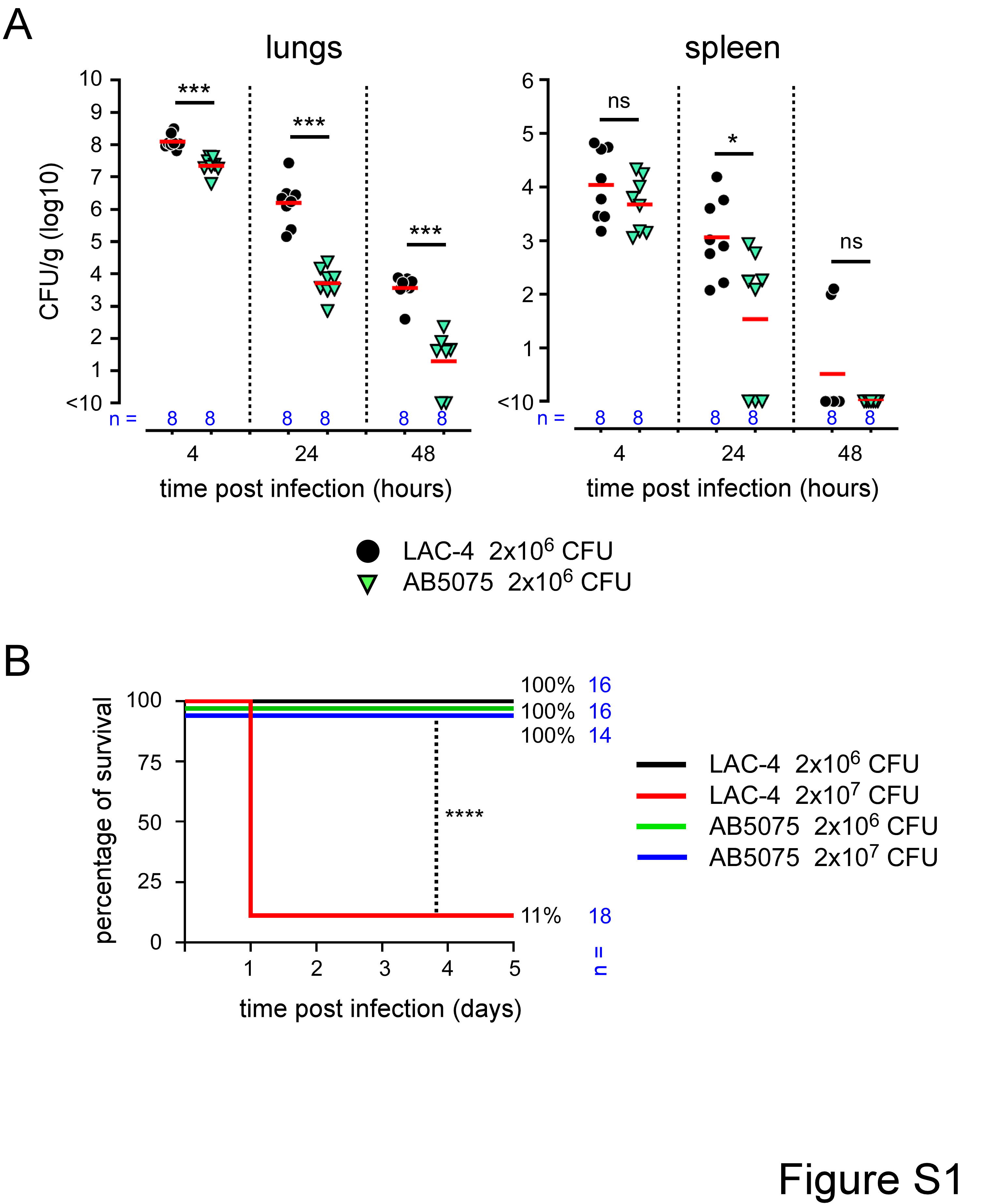

Supplement: Supplementary Figure 1 — Comparison of the virulence of LAC-4 and AB5075 strains in the C57BL/6 mouse intranasal infection model. Wild-type C57BL/6 mice were inoculated intranasally (IN) with 2x10^6^ or 2x10^6^ CFU of LAC-4 or HK 5075 A. baumannii strains. (A) At 4, 24 and 48 hours post infection, some mice were sacrificed and the number of bacteria per gram in the lungs and spleen was evaluated by CFU counting. Red lines represent the geometric mean for each group of mice. These data are representative of two independent experiments. (B) During the 120 hours following infection, the fitness of infected mice was monitored. When the human endpoint was reached, the mice were euthanized. These data represent a pool of two independent experiments. n = number of mice per group. Significant differences between groups are marked with asterisks: *p < 0.05, ***p < 0.001, ****p < 0.0001, in a log-rank (Mantel–Cox) test for survival curve and a (Wilcoxon-) Mann-Whitney post-test for CFU count. [file Image1.tif]

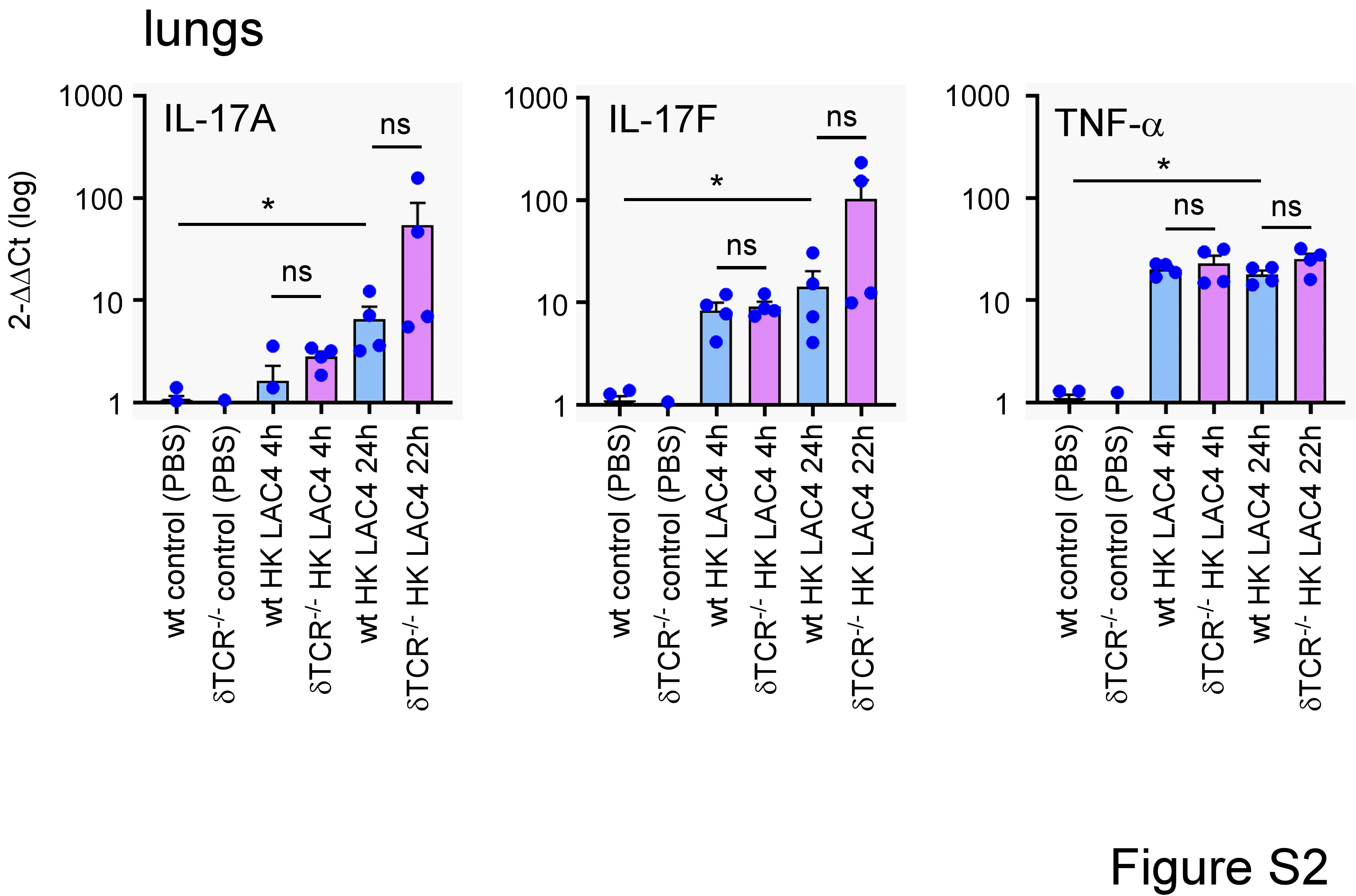

Supplement: Supplementary Figure 2 — Production of IL-17A, IL-17F, IL-22, and TNF-α is not reduced in δTCR-deficient mice in response to infection with A. baumannii LAC-4 strain. Wild-type and δTCR-/- C57BL/6 mice were inoculated intranasally with PBS (control) or 2x10^6^ CFU of live A. baumannii LAC-4 strain in PBS, as indicated. At 4 and 24 hours post-infection, mice were sacrificed, and lungs were collected. Expression levels of the indicated genes were assessed by quantitative RT-PCR and expressed as relative expression to RPL32 housekeeping mRNA (log10). The naïve mice (number 1) condition was set to 1. n = 4 mice for each condition. Significant differences between groups (B) are marked with asterisks: ****p < 0.0001, in a (Wilcoxon-) Mann-Whitney post-test. [file Image2.tif]

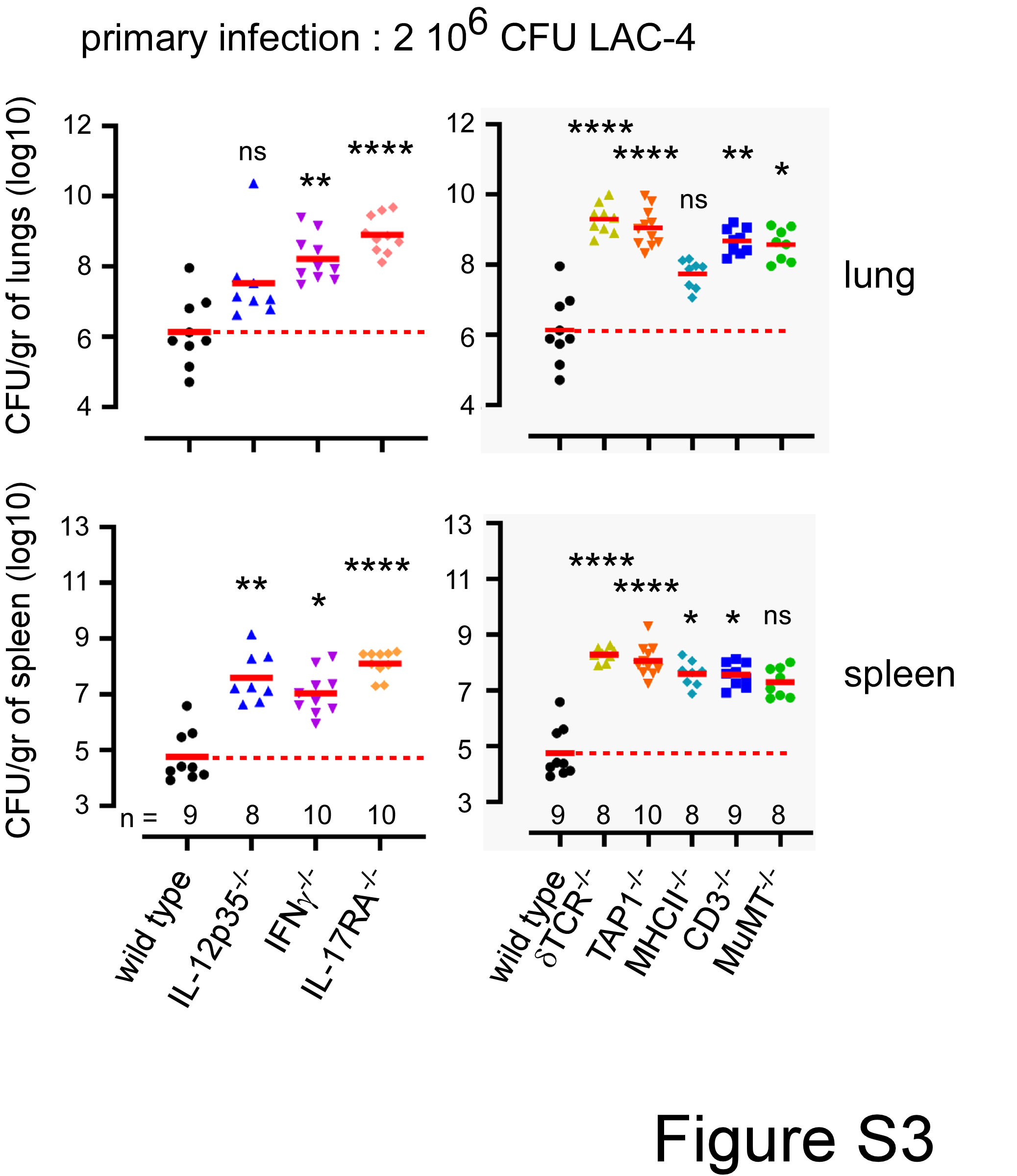

Supplement: Supplementary Figure 3 — Evaluation of A. baumannii LAC-4 infection control in different stains of C57BL/6 mice. Wild-type and immunodeficient C57BL/6 mice were infected intranasally with LAC-4 A. baumannii (2.106 CFU) and sacrificed at 24 hours post-infection. The data represent the number of CFU/g of lungs or spleen, as indicated. Red lines represent the geometric mean. Dotted red line represents the mean of the results for wild-type mice. Significant differences between wild-type and deficient mice are marked with asterisks: *p < 0.1, **p < 0.01, ***p < 0.001, ****p < 0.0001, in a (Wilcoxon-) Mann-Whitney post-test. [file Image3.tif]
